# Non-invasive Ischaemia Testing in Patients With Prior Coronary Artery Bypass Graft Surgery: Technical Challenges, Limitations, and Future Directions

**DOI:** 10.3389/fcvm.2021.795195

**Published:** 2021-12-23

**Authors:** Andreas Seraphim, Kristopher D. Knott, Joao B. Augusto, Katia Menacho, Sara Tyebally, Benjamin Dowsing, Sanjeev Bhattacharyya, Leon J. Menezes, Daniel A. Jones, Rakesh Uppal, James C. Moon, Charlotte Manisty

**Affiliations:** ^1^Department of Cardiac Imaging, Barts Health National Health System Trust, London, United Kingdom; ^2^Institute of Cardiovascular Science, University College London, London, United Kingdom; ^3^William Harvey Research Institute, Queen Mary University of London, London, United Kingdom

**Keywords:** CABG, ischaemia detection, surgical revascularisation, stress imaging, myocardial perfusion

## Abstract

Coronary artery bypass graft (CABG) surgery effectively relieves symptoms and improves outcomes. However, patients undergoing CABG surgery typically have advanced coronary atherosclerotic disease and remain at high risk for symptom recurrence and adverse events. Functional non-invasive testing for ischaemia is commonly used as a gatekeeper for invasive coronary and graft angiography, and for guiding subsequent revascularisation decisions. However, performing and interpreting non-invasive ischaemia testing in patients post CABG is challenging, irrespective of the imaging modality used. Multiple factors including advanced multi-vessel native vessel disease, variability in coronary hemodynamics post-surgery, differences in graft lengths and vasomotor properties, and complex myocardial scar morphology are only some of the pathophysiological mechanisms that complicate ischaemia evaluation in this patient population. Systematic assessment of the impact of these challenges in relation to each imaging modality may help optimize diagnostic test selection by incorporating clinical information and individual patient characteristics. At the same time, recent technological advances in cardiac imaging including improvements in image quality, wider availability of quantitative techniques for measuring myocardial blood flow and the introduction of artificial intelligence-based approaches for image analysis offer the opportunity to re-evaluate the value of ischaemia testing, providing new insights into the pathophysiological processes that determine outcomes in this patient population.

## Introduction

Coronary artery bypass surgery is the most frequently performed cardiac surgical procedure, with ~200,000 patients undergoing isolated coronary artery bypass surgery each year in the US (1). Despite improved post-operative survival (2) particularly among high risk groups (3, 4), patients undergoing surgical revascularisation represent the severe end of coronary artery disease spectrum and comprise a high risk group. With long term survival of patients undergoing CABG approaching that of the general population (5–7), a significant number of patients with prior CABG surgery are expected to experience symptom recurrence requiring re-intervention (8). Studies have reported considerable rates of myocardial infarction and ischaemia-driven revascularisation even within the first 5-years post CABG (9). Data from the European Heart Survey suggests that 14% of patients undergoing coronary revascularisation between 2001 and 2002 had had a history of CABG (10), with similar rates seen in more contemporary data from the UK (11). Importantly, outcomes following repeat revascularisation are significantly worse compared to patients with no history of CABG, both in the context of stable coronary artery disease (11) and acute coronary syndromes (12). It is therefore not surprising that there is a clinically-driven, high demand for detailed functional non-invasive investigations for myocardial ischaemia in this patient group.

## Coronary and Graft Disease Post Coronary Artery Bypass Graft Surgery

The two key pathophysiological processes thought to be driving symptom recurrence are vein graft failure and progression of native vessel coronary disease. Graft failure after coronary artery bypass graft surgery is thought to follow a bimodal distribution, often defined as early (<6 months) or late (13), and is known to be higher for venous compared to arterial grafts (5, 14). Vein graft failure (VGF) rates of up to 25% during the first 12–18 months post CABG are reported even in contemporary studies (15, 16), with VGF rates of 40–50% seen at 10 years (17). In contrast, internal mammary grafts have a reported 10-year patency rate over 90% (18). At the same time, native disease progression appears to accelerate particularly in bypassed vessels, with up to 46% new total occlusions seen within 5 years post CABG (19). Although data on the prognostic impact of graft failure is conflicting (20–22), both graft failure and native disease progression are associated with symptom recurrence, and are often the suspected processes prompting evaluation of ischaemia.

Invasive coronary and graft angiography remains the definitive anatomical test for evaluating the extent of coronary disease, but may not provide sufficient information to guide complex management decisions post CABG due to the lack of functional correlation. Physiological lesion assessment using fractional flow reserve (FFR) is more challenging in the context of grafts, due to the severity and complexity of native coronary artery disease (calcification, tortuosity, and chronic total occlusions) and the differing flow relationships between native and graft circulations. Therefore, clinical decision-making based on FFR warrants caution (23), as although technically feasible there is limited data to support its use. Importantly, given the different physiological profile of vein grafts compared to native coronary vessels with regards to the rate of disease progression, extrapolating findings of major clinical trials (24) demonstrating the clinical utility of FFR assessment in native vessels which tended to exclude post CABG patients may be inappropriate. Furthermore, alterations in native coronary anatomy and the interplay between extensive diffuse disease and development of collateral systems result in unique and complex haemodynamic circuits that are difficult to evaluate (25). Detection of ischaemia in the context of chronic coronary artery disease is therefore often performed using a range of non-invasive imaging tests, and represents a large proportion of cardiac investigations, resulting in significant healthcare costs (26). Both the United Kingdom National Institute of Clinical Excellence (NICE) (27) and European Society of Cardiology guidelines (28) advocate the use of a non-invasive functional testing for evaluation of patients with known coronary artery disease, including those with previous revascularisation.

## Current Guideline Recommendations and Challenges for Imaging Post CABG

All non-invasive imaging modalities have technical limitations in terms of image acquisition and interpretation, affecting their diagnostic performance. The challenges associated with the use of non-invasive ischaemia evaluation of patients with prior CABG are indeed reflected by a degree of discrepancy among societal guidelines. For example, according to American College of Cardiology (ACC) Task force recommendations, the use of stress echocardiography in asymptomatic patients solely for the purpose of risk stratification is not recommended within 5 years from CABG surgery (29). In contrast, the more recent European Society of Cardiology (ESC) guidelines are more liberal in the use of non-invasive testing post revascularisation, even supporting the use of early ischaemia testing for setting a reference, or periodically every 3–5 years (28). In terms of symptomatic patients with prior CABG, the American College of Cardiology/American Heart Association (ACC/AHA) guidelines recommend evaluation using non-invasive stress imaging tests, with a preference toward exercise as a method of stress (30). Similarly, the European guidelines recommend the use of stress imaging over exercise stress ECG if practically possible (28). Beyond this, international guidelines offer little guidance on the choice of functional testing after CABG, resulting in wide variations in practice patterns. Indeed, large multicentre registry data confirm that the choice of stress testing after CABG is primarily defined by the clinical center rather than patient clinical characteristics (31).

Both detection and interpretation of ischaemia testing in patients with CABG remains a challenge, and despite the availability of a wide array of diagnostic imaging tools no test has demonstrated superiority in these patients. Difficulties associated with ischaemia testing in patients following surgical revascularisation are primarily due to the complex anatomical, haemodynamic and myocardial alterations that result from surgery. Surgery results in variable degrees of electro-mechanical myocardial abnormalities (32, 33) and changes in coronary anatomy and flow (34), often limiting the diagnostic performance of all non-invasive tests. Consequently, questions remain as to whether any functional imaging test is sensitive and specific enough to identify subtle differences in regional myocardial blood flow or contractility and provide reliable data to inform revascularisation strategies and appropriately risk stratify patients. As any form of revascularisation post coronary artery bypass is associated with increased risk of complications (35, 36) and suboptimal outcomes (37, 38), accurate detection and consistent interpretation of ischaemia becomes important. Furthermore, accepting the notion that the extent of myocardial ischaemia translates to a higher risk of future ischaemic events, and considering the poor outcomes after myocardial infarction in this patient group (39), accurate detection of ischaemia in these patients may enable risk stratification and potentially guide clinical management.

## Challenges in Non-invasive Stress Testing Post-surgical Revascularisation

Patients referred for CABG surgery typically have advanced epicardial disease, which is often a combination of both focal and diffuse atherosclerosis involving multiple coronary territories. This is also a reflection of a higher risk population that suffers with significant comorbidity, which further complicates ischaemia testing. For example, left ventricular (LV) dysfunction is not uncommon in patients post-surgical revascularisation, and this poses additional challenges in the evaluation of ischaemia beyond the complexity of the underlying coronary artery disease. The degree of LV impairment is thought to impact the stress response to pharmacological vasodilators such as adenosine, with prior studies suggesting that increased dosing may be needed in these patients (40). Similarly, the presence of LV dysfunction increases arrhythmic complications in those undergoing dobutamine stress testing (41), whereas pharmacological therapy such as beta-blockers may blunt heart rate augmentation during exercise. Beyond the haemodynamic effects of LV dysfunction, the presence of implantable electronic devices can be detrimental to image quality and the ability to elicit adequate heart rate response during stress. The high prevalence of cardiovascular factors that contribute to the development of coronary artery disease also increase the risk of chronic kidney and lung disease (42), introducing additional limitations in the use of each imaging modality. Furthermore, additional challenges related to the complexity of post-CABG coronary and myocardial blood flow physiology, as well as technicalities in image acquisition further complicate ischaemia assessment.

### Challenges in Evaluating Myocardial Blood Flow Post Coronary Artery Bypass Graft Surgery

#### Coronary Anatomy and Myocardial Ischaemia Correlation

**Figure 1:**
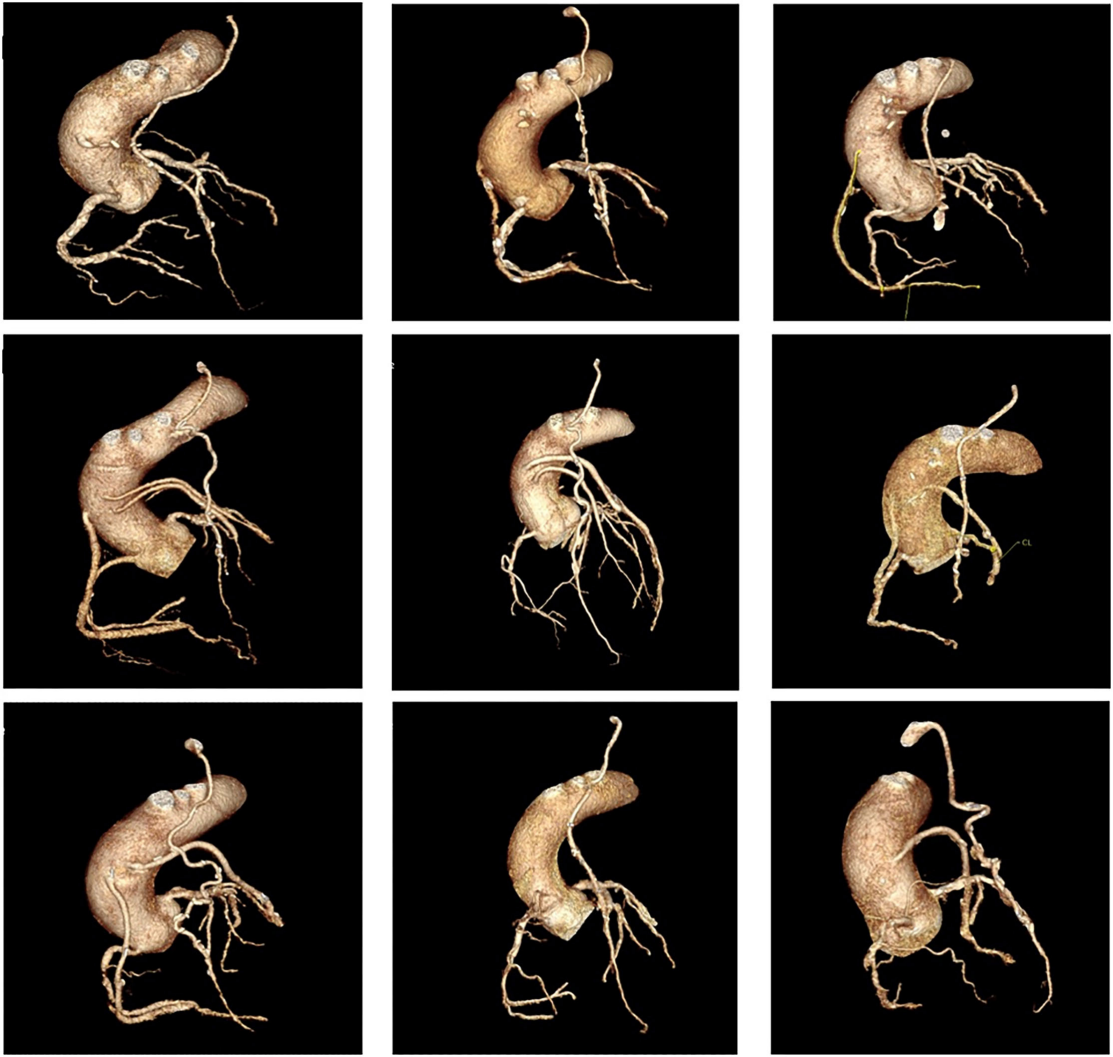
Panel of nine cases of coronary anatomy post coronary artery bypass graft surgery. All cases show a left internal mammary artery anastomosed to the to the left anterior descending artery (LAD), demonstrating significant variations in anastomosis position along the length of the vessel, as well as significant variations in the post-operative anatomy of the remaining vessels.

Coronary artery bypass graft surgery results in significant alterations in coronary physiology, with significant variations in post-operative anatomy among individual patients. Variations in anastomosis position and complex grafting approaches based on the distribution and severity of native vessel disease mean that correlation of ischaemia and coronary anatomy is often difficult in patients following surgery (Figure 1).

**Figure 2:**
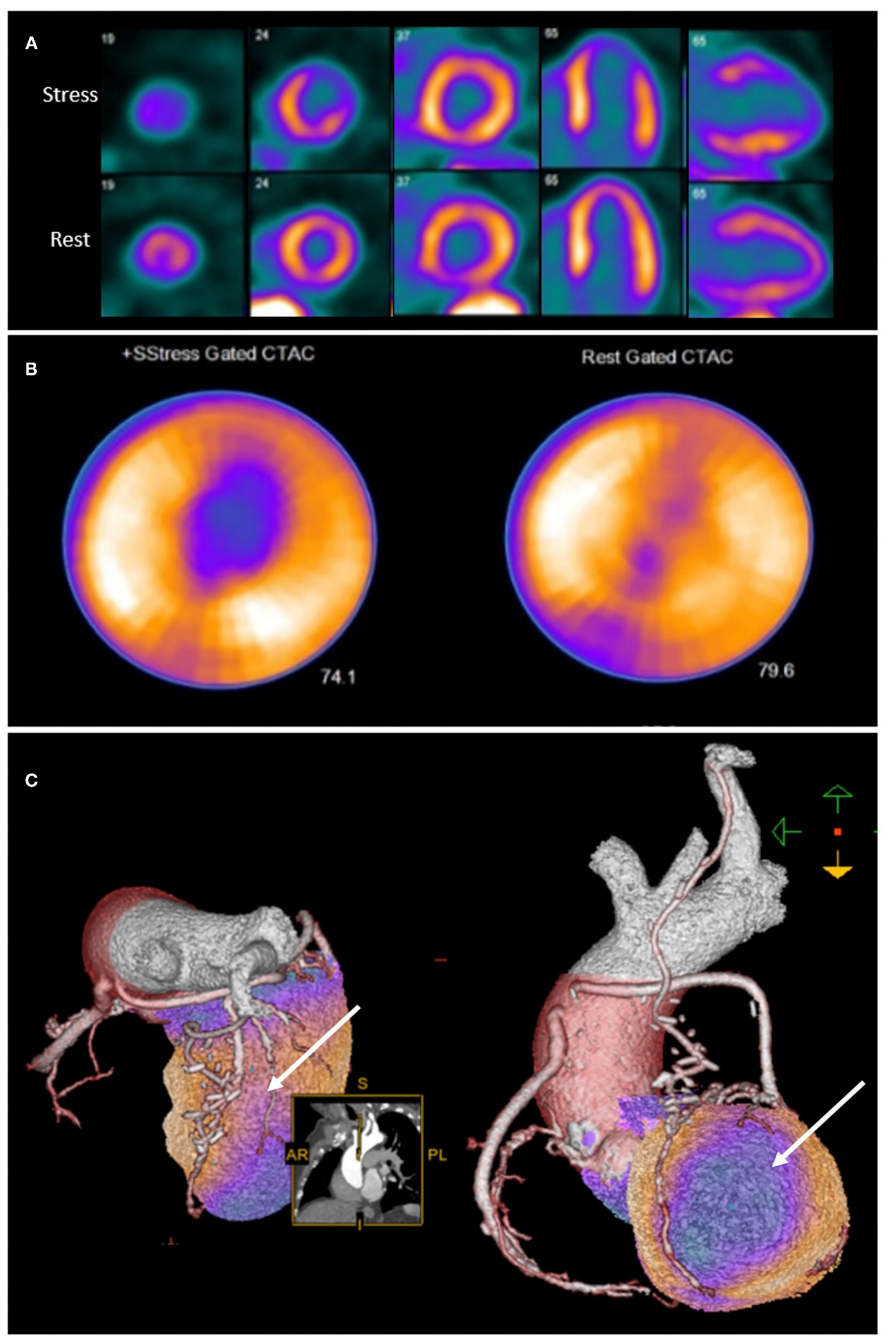
Rubidium-82 PET-CT with adenosine stress in an 86-year-old male with previous coronary artery bypass grafting. PET-CT images **(A,B)** obtained at stress and rest demonstrate a reversible perfusion defect in the mid to apical anterior segments extending into the apex. Cardiac hybrid imaging with three-dimensional fusion of PET-CT with CT coronary angiography enables localization of ischaemia to a coronary artery territory **(C)**. CT coronary angiography reveals a patent LIMA to LAD graft with good distal opacification, and obstructive plaques in the proximal and mid segments of an intermediate artery (white arrow), responsible for the reversible perfusion defect demonstrated.

Integration of anatomical and perfusion data may therefore facilitate re-assignment of ischaemia to culprit vessel, and may provide additional insights into the mechanism of ischaemia (43). It is therefore unsurprising that hybrid imaging techniques that combine anatomical and functional testing in patients post CABG provide incremental information on the localization of atherosclerotic lesions (43) as well as prognosis (44) (Figure 2).

**Figure 3:**
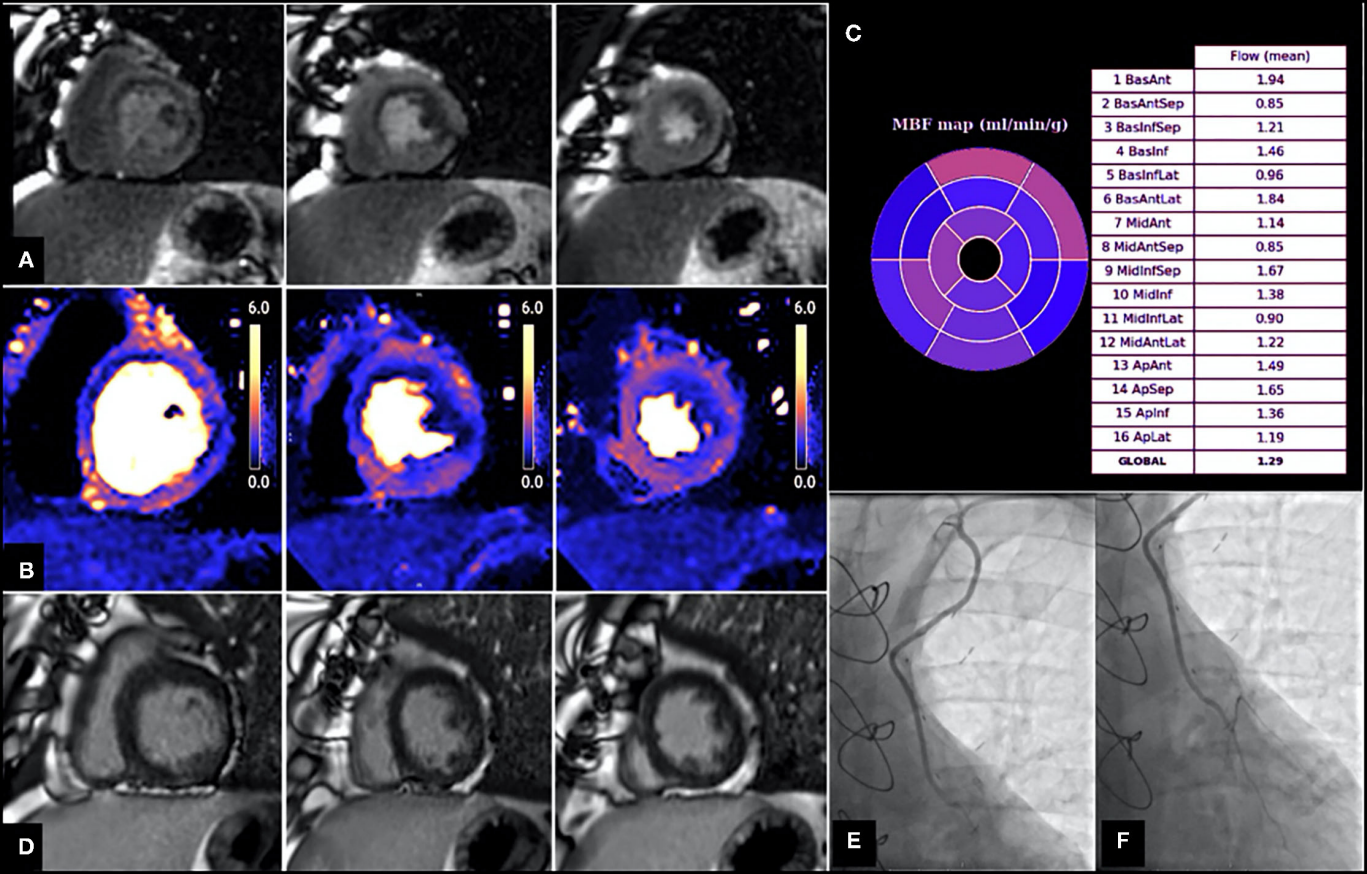
Patient with angiographically confirmed patent LIMA to LAD and evidence of inducible perfusion defect in LIMA—native LAD subtended territories. Images shown are short axis views from base to apex (left to right). **(A)** First pass perfusion imaging with adenosine stress, demonstrating a perfusion defect in the basal to mid antero-septum, basal to mid anterolateral and inferolateral and apical lateral walls. **(B)** Stress myocardial blood flow (MBF) evaluation using perfusion CMR showing reduced MBF in multiple territories, including those supplied by the LIMA—native LAD (e.g., MBF in mid antero-septum is 0.85 ml/g/min, MBF in apical septum is 1.65 ml/g/min). **(C)** Bullseye plot demonstrating stress MBF in each myocardial territory based on American Heart Association (AHA) segmentation. **(D)** Late gadolinium images showing no evidence of previous infarction in the LIMA—native LAD territories. **(E,F)** Coronary angiography demonstrating patent LIMA graft **(E)** and anastomosis site **(F)** with good distal run off. From Seraphim et al. (51). Reproduced under the Creative Commons Attribution 4.0 International License.

However, the extent of coronary artery disease encountered in these patients often promotes the development of extensive collateral systems (45) and these can often complicate correlation of ischaemia to a corresponding epicardial vessel. Similarly, CABG surgery often results in unique haemodynamic conditions such as retrograde and competitive blood flow (46) that cannot be easily characterized by non-invasive tests. A number of studies evaluating stress myocardial perfusion post CABG reported a high prevalence of perfusion defects in territories supplied by patent grafts (43, 47–50), however the underlying mechanism of this remains unclear (Figure 3). Discrepancy between graft and native vessel size (47), persistent microvascular dysfunction (52), technical limitations associated with delayed contrast arrival (53) and native coronary artery disease progression either proximal or distal to the anastomosis (48, 51) have all been proposed as potential contributing mechanisms. Data from the SWEDEHEART registry suggested that a substantial amount of invasive coronary angiography performed due to symptom recurrence identified no graft failure, highlighting the possibility that symptom recurrence post CABG may be largely attributed to native disease progression (54). However, despite the frequency of such perfusion defects in patients post CABG, interpretation and subsequent management varies considerably between clinicians.

#### Myocardial Infarction and Evaluation of Peri-Infarct Ischaemia

**Figure 4:**
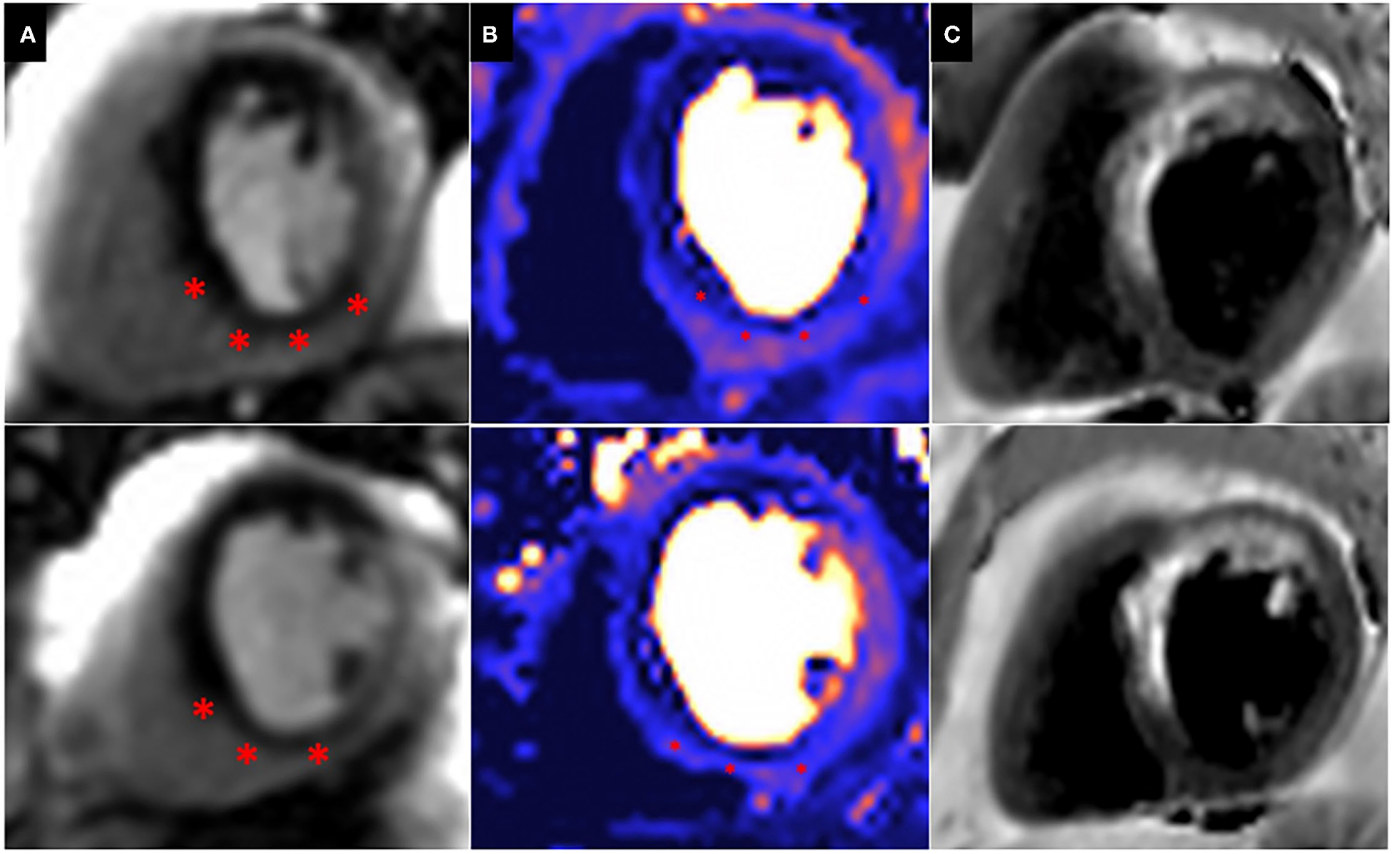
Peri-infarct ischaemia and scar. Basal (top) and Mid (Bottom) short axis views of a CMR perfusion in patient scheduled to undergo coronary artery bypass graft surgery, demonstrating a previous infarct within the left anterior descending (LAD) territory and a large superimposed perfusion defect extending beyond the area of previous infarction (^*^). **(A)** First pass perfusion CMR during adenosine stress; **(B)** Perfusion mapping of the same myocardial segment as shown in **(A)**. **(C)** Dark blood LGE demonstrating a previous infarction within the LAD territory.

Patients with prior surgical revascularisation often have complex patterns of previous myocardial infarction with a number of studies demonstrating a wide range of scar pattern and distribution post procedure (55, 56). This indeed reflects the multi-factorial etiology of ischaemic injury sustained by these patients, including the impact of surgery itself. The presence of complex myocardial scar makes evaluation of ischaemia challenging, particularly when this is super-imposed or adjacent to areas of scar. Bernhardt et al., combined CMR perfusion and tissue characterization with late gadolinium enhancement (LGE) assessment and reported improved prediction of clinically relevant bypass graft stenosis, supporting the idea that ischaemia interpretation in patients post CABG requires some knowledge of scar distribution (57) (Figure 4).

**Figure 5:**
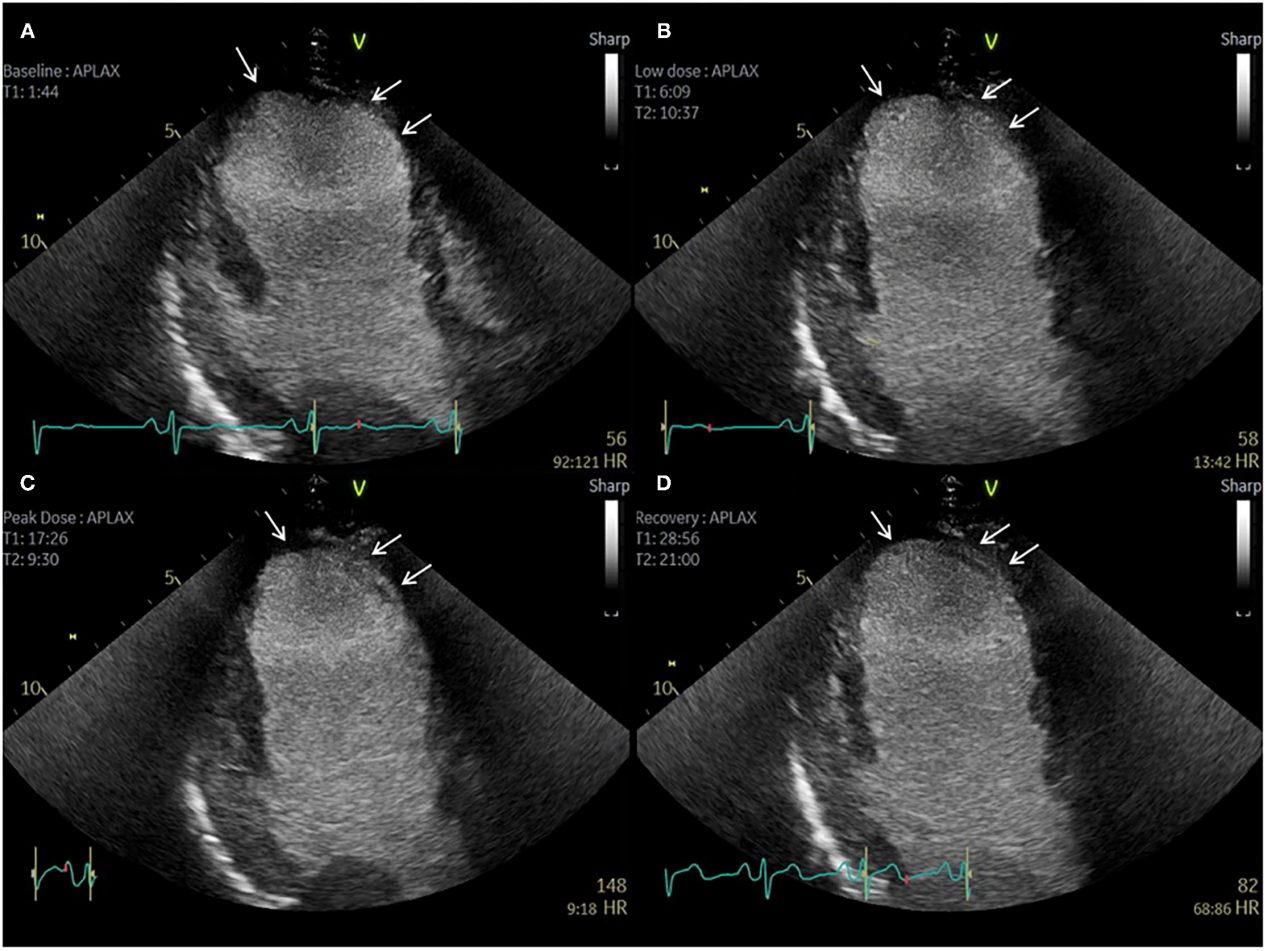
Contrast echocardiography post coronary artery bypass graft surgery. Sixty-three-year-old patient with previous CABG and atypical chest pain. **(A–D)** Apical 3 Chamber view. Baseline, low dose, peak dose, and recovery stages, respectively. Contrast Enhanced Images. Akinetic mid and apical antero-septal wall segments (arrow). No improvement in contractility of these segments during low dose stage confirms non-viable segments. Improvement in contractility of all other segments during low dose and peak dose suggests the presence of viable myocardium and no inducible ischaemia.

Evidently, imaging modalities that can provide simultaneous evaluation of ischaemia and tissue characterization can be advantageous in these circumstances, more so in cases of extensive or complex anatomical scar. Similarly, imaging modalities capable of providing complete LV coverage such as PET and SPECT enable a more comprehensive assessment of the relation between ischaemia and scar in this context, particularly when paired with anatomical data (Figure 2). Despite this, echocardiography remains the most widely used modality for evaluation of relative differences in wall motion, and often enables accurate evaluation of the extent of regional viability and ischaemia, particularly when facilitated by contrast echocardiography (Figure 5) (58). It is worth noting that a recent expert consensus statement on the use of multimodality of myocardial viability, makes no recommendations on a preferred imaging modality in this population (59), further highlighting the complexities in the evaluation of patients with prior CABG.

#### Incomplete Revascularisation at the Time of Surgery

One of the key aims of coronary artery bypass graft surgery is to minimize myocardial ischaemia through complete revascularisation if this is technically attainable (60). However, native vessel characteristics such as heavy calcification and small vessel size, often result in modification of the revascularisation strategy intra-operatively, with a number of myocardial territories remaining un-grafted (61). In a meta-analysis of 25,938 patients undergoing CABG surgery, Garcia and colleagues (62) reported that incomplete revascularisation was detected in 25% of patients. Beyond this, even if complete anatomical bypassing of significant epicardial coronary lesions is performed, restoration of coronary flow using grafts is unlikely to accurately replicate native coronary disease flow and haemodynamic conditions. Indeed, studies evaluating myocardial blood flow shortly after CABG surgery, reported that myocardial blood flow remains lower than commonly reported values in patients with native vessel disease (Table 1). Although arguably a reflection of more advanced coronary artery disease, it is conceivable that in a significant proportion of patients post CABG, some degree of ischaemia is often encountered despite successful surgical revascularisation.

**Table 1:** Non-invasive myocardial blood flow assessment post-surgical revascularisation.

**References**	**Modality**	**Number of patients**	**Indication for perfusion assessment**	**Time from CABG**	Stress MBF^*^	MPR^*^
**Myocardial blood flow post CABG**						
Aikawa et al. (63)	^15^O-water PET	47	Protocol-driven assessment	6 months	1.45 (1.27–1.88)	1.93 (1.64–2.56)
Driessen et al. (64)	^15^O-water PET	18	Protocol-driven assessment	62 days	2.05 ± 0.65	2.63 ± 0.87
Seraphim et al. (51)	Adenosine stress CMR	38	Clinical indication for scan; patent LIMA grafts	5 years	1.54 ± 0.47	1.94 ± 0.63
Spyrou et al. (52)	^15^O-water PET	8	Protocol-driven assessment	6 months	2.45 ± 0.64	2.57 ± 0.49
**Healthy controls**						
Gould et al. (65)	PET (different tracers)	3,482	Healthy controls	n/a	2.86 ± 1.29	3.55 ± 1.36
Brown et al. (66)	Adenosine stress CMR	42	Healthy controls	n/a	2.71 ± 0.61	4.24 ± 0.69
Zorach et al. (67)	Regadenoson stress CMR	20	Healthy controls	n/a	3.17 ± 0.49	2.93 (2.76–3.19)

*MBF, myocardial blood flow; MRP, myocardial perfusion reserve; LIMA, left internal mammary artery*.

* *Results presented as median (inter-quartile range) or mean ± standard deviation*.

Differentiating graft failure or progression of native coronary disease from incompletely revascularised myocardium is challenging, especially without some form of early post-operative evaluation as a baseline. Indeed, the latest ESC guidelines on chronic coronary syndromes (28) recommend the use of non-invasive ischaemia evaluation for documentation of residual ischaemia as a reference for subsequent assessment.

#### Microvascular Ischaemia

Myocardial blood flow following surgical revascularisation is not solely governed by native epicardial coronary disease. Microvascular disease, is almost universal in patients post CABG, and is also associated with a reduction in stress MBF and perfusion reserve (MPR) (68). Itself an independent predictor of outcomes in patients with native vessel disease (69), the clinical consequences of microvascular disease in patients with prior CABG are poorly understood. Our understanding of the physiological impact of surgery itself, particularly the effect of cardioplegic arrest and cardiopulmonary bypass (70) on microvascular function is limited, with both invasive and non-invasive studies reporting an early post-operative impairment of flow that appears to recover over time (52, 71). Importantly, the impact of surgery may differ among subgroups, with some evidence suggesting that patients with diabetes experience worse microvascular dysfunction post-operatively (72). Advances in image quality across all modalities has meant that non-invasive tests are becoming increasingly capable of detecting microvascular disease, thereby providing additional insights into the pathophysiological mechanisms of reduced myocardial blood flow. Quantitative perfusion indices such as stress MBF and MPR have been used to help differentiate epicardial coronary disease and microvascular dysfunction in the context of native vessel disease (73), but whether a similar assessment can be performed in patients with prior surgical revascularisation is unclear.

### Technical Challenges in Non-invasive Stress Imaging in Patients With Prior CABG

#### The Effect of Prolonged Contrast Transit Time in Graft Subtended Myocardial Territories

All tracer-based methods of ischaemia evaluation, rely on the peripheral injection of an intravenous contrast or tracer and the subsequent acquisition of a dynamic series of myocardial images. Subsequent use of tracer-specific kinetic models allows quantification of myocardial blood flow (MBF) (74). In patients with prior CABG, the increased length of graft conduits results in a prolonged tracer transit time, potentially distorting the first pass kinetics of the contrast bolus complicating both the visual interpretation of relative perfusion defects and the subsequent estimation of myocardial blood flow in graft-subtended territories (75). Such delay in contrast arrival, although small, is thought to particularly affect longer conduits, such as internal mammary (LIMA) grafts (53). Very few studies evaluated the effects of delayed transit of contrast in grafts, with conflicting results in terms of its absolute impact on quantitative indices of myocardial perfusion (51, 53). Data using computational fluid dynamics modeling reveals a close relationship between local coronary hemodynamics and contrast dispersion that potentially impacts any bolus-based perfusion measurement (76, 77). It is therefore possible, that all tracer kinetic modeling methods used to estimate MBF would need to consider the effects of differential contrast arrival, presence of collateral flow and blood mixing from competitive flow as possible sources of systematic error of quantitative blood flow measurements.

#### Differential Response to Pharmacological Stress Between Vein vs. Arterial Grafts

Most non-invasive tests for ischaemia rely on the detection of relative perfusion imbalances caused by a differential hyperaemic response to some form of pharmacological challenge. The effect of a number of vasoactive agents such as adenosine, dipyridamole and regadenoson (78), on the native coronary circulation is to a certain degree predictable and reproducible (66), and this simplifies their use as pharmacological stressors. However, the possibility of a differential vasoactive effect on grafts, particularly a disparity between arterial and venous grafts raises concerns with regards to the use of these agents in patients post CABG.

Previous studies using invasive haemodynamic data showed a reduced, or indeed absent, vasodilatory response of venous compared to arterial grafts following intra-graft injection of adenosine (79, 80). Similar findings were obtained with other pharmacological vasodilators (81). Indeed, these differences in vasomotor properties between graft conduits have been proposed as a possible explanation for the variability in long-term patency between arterial and venous grafts (82). Whether this differential response to pharmacological stress limits the diagnostic accuracy of perfusion detects remains unclear. Arnold et al. demonstrated that the hyperaemic MBF in response to adenosine was higher in segments supplied by arterial compared to venous grafts as assessed by quantitative CMR (83), further highlighting that quantitative ranges and cut offs for defining normal myocardial blood flow and myocardial perfusion reserve may differ from those seen in native coronaries. Whether the use of alternative pharmacological stress agents such as dobutamine would overcome this potential limitation is unclear. Dobutamine increases myocardial blood flow predominantly through an increase in myocardial oxygen demand, resulting from the increase in heart rate and myocardial contractility (84), although it is also thought to exert a relative weaker direct vasodilatation effect (85). Limited data exist on head to head comparison of different stress agents in patients post CABG (86, 87), but superiority of dobutamine over commonly used stressors has not been confirmed. Data from patients without previous surgical revascularisation would suggest that coronary flow augmentation is significantly higher with vasodilator agents such as adenosine and regadenoson compared to dobutamine or exercise, but whether this translates to an improved diagnostic performance is unclear. Furthermore, comparison between exercise and pharmacological stressors has not been widely studied in the context of previous CABG (88).

#### Arrhythmia and Electro-Mechanical Changes Post CABG

Surgical revascularisation results in both electrical and myocardial structural changes (89), thought to be secondary to procedural-related factors such as shifts in myocardial position (90), pericardial release (91), and peri-operative ischaemic injury (92). Atrial arrhythmias, particularly atrial fibrillation, are also common after surgery (93) and these are known to impact on the diagnostic performance of essentially all non-invasive ischaemia tests. Abnormal septal motion is common after cardiac surgery (94), making the interpretation of wall motion evaluation at both rest and peak stress challenging. Similarly, the electro-mechanical response to pharmacological agents such as dobutamine is thought to be altered in patients following CABG (95).

## Diagnostic Performance of Non-invasive Ischaemia Testing for the Detection of Graft Failure and Native Disease Progression

Ischaemia testing post-surgical revascularisation is broadly performed to evaluate distinct pathophysiological processes, which if identified can potentially alter clinical management. These include the presence of graft failure, native disease progression and in some cases the presence of residual ischaemia when incomplete revascularisation is suspected.

Each imaging modality suffers from its own limitations (Table 2) when it comes to surgically treated patients and in most studies the reported performance is inferior to that seen in patients without prior CABG (57, 96). Echocardiography is the most widely used technique for ischaemia evaluation and its diagnostic accuracy has been reported in a number of studies, using both pharmacological and exercise testing (97–102). In the context of CABG, limited LV coverage, and challenges in visualizing viable myocardium, peri-infarct ischaemia, and detecting multi-vessel disease are the main limitations. Although myocardial contrast echocardiography can overcome some of these limitations by offering quantitative perfusion assessment (103), it has not gained wider acceptance clinically, mainly due to lack of automation that hinders its adoption into the clinical workflow. CMR is being increasingly described as a reproducible and accurate method of ischaemia assessment, with an expanding body of evidence confirming its prognostic value and cost-effectiveness in the context of native coronary artery disease (104, 105). However, all major studies evaluating the diagnostic and prognostic performance of stress perfusion CMR have excluded patients with prior CABG, reflecting the complexity of ischaemia assessment in this patient population. Limited LV coverage with CMR poses challenges, particularly in terms of co-registering coronary anatomy and perfusion assessment and as in all modalities depending on first pass perfusion, there are questions regarding the impact of arterial delay of contrast through long grafts (51, 75). Despite increasing evidence on the safety of CMR imaging in patients with implantable electronic devices (106), artifact can affect image quality and perfusion interpretation. Furthermore, cost and limited availability continue to impede its clinical adoption as a mainstream test for ischaemia evaluation. Despite this, due to its high spatial resolution CMR is well-suited for evaluation of peri-infarct ischaemia and viability assessment in the same setting. Nuclear techniques (both PET and SPECT), have historically been crucial non-invasive modalities for ischaemia testing, and their performance and prognostic use is supported by a large body of evidence (22, 88, 107–112). As for native disease assessment, exposure to ionizing radiation continues to be considered a limitation, although with novel cameras and tracer technology the dose of this is decreasing. Furthermore, the possibility of hybrid imaging with computed tomography (CT) offers a great potential, with the advantage of paired anatomical and perfusion analysis being particularly relevant in the context of prior surgical revascularisation. Computed tomography coronary angiography itself offers an excellent tool for anatomical evaluation of graft patency (113), with high diagnostic accuracy for detection of graft occlusion or stenosis, but heavy calcification and native vessel disease especially in anastomotic sites and small-caliber distal runoff vessels, reduce its overall diagnostic performance without the benefit of paired perfusion assessment. Beyond this, mathematically modeled fractional flow reserve using CT (FFR_CT_) has not been validated among patients with prior CABG (114) and its use in this patient population is not currently recommended (115).

**Table 2:** Comparison of non-invasive imaging tests for the assessment of myocardial ischaemia in patients with previous coronary artery bypass grafts—features, strengths, and limitations.

**Imaging modality**	**Stressor**	**Accessibility/risks**	**Ischaemia / perfusion**	**Viability and function**	**Coronary anatomy**	**Quantitative perfusion**
Stress echo	• Exercise, dobutamine, vasodilator	• Widely available • Often requires use of contrast for image quality • No radiation • Risk associated with dobutamine in the context of LV dysfunction	• Limited LV coverage • Less sensitive to identify subtle RWMA • Arrhythmia and abnormal septal motion limit performance • Spatial resolution: 1 × 1–3 × 3–6 mm^3^	• Viability assessment suboptimal compared to CMR and PET	• N/A	• Requires use of microbubbles and associated with technical challenges • Linear relationship between blood flow and tracer
CMR	• Mainly vasodilator • Dobutamine and exercise possible but limited	• Not widely available • Vendor, field strength, sequence differences • No radiation • Devices affect image quality	• Limited LV coverage (conventionally 3x short axis slices used) • Arrhythmia can be detrimental • Can identify peri-infarct ischaemia • Spatial resolution: 1 × 2 × 6–8 mm^3^	• Gold standard modality for volume assessment • Peri-infarct ischaemia assessment • Additional tissue characterization	• Not performed routinely • Limited LV coverage	• Altered contrast kinetics associated with complex graft-native vessel flow • Non-linearity between blood flow, contrast and signal intensity
SPECT	• Exercise or vasodilator	• Widely available • Radiation (significantly reduced with modern scanners)	• Isotropic left ventricle coverage • Limited spatial resolution: 10 × 10 × 10 mm^3^	• Viability and function assessment possible • Limited temporal resolution	• Hybrid imaging with CT possible	• Limited temporal resolution • New generation scanners offer quantitative analysis
PET	• Exercise or vasodilator	• Not widely available • Radiation	• Isotropic left ventricle coverage • Endocardial- epicardial flow estimation possible • Spatial resolution 4 × 4 × 4 mm^3^	• Viability assessment possible • Lower spatial resolution than CMR	• Hybrid imaging with CT possible	• Linear relationship between blood flow and ^15^O-water • Linear relationship between tracer and image signal
CT perfusion/ angiography	• Vasodilator	• Perfusion not widely available • Radiation	• Spatial resolution (image analysis): 0.5 × 0.5 × 6–8 mm^3^ • Isotropic left ventricle coverage • Low CNR • Coronary and graft anatomy available	• Viability and function assessment possible, but increased radiation dose	• Data on anatomy • Difficulties with anastomosis sites and natives. • CT-FFR not validated for patients post CABG	• Non-linear relationship between blood flow and contrast

*SPECT, Single-Photon Emission Computed Tomography; CMR, Cardiac Magnetic Resonance; PET, Positron Emission Tomography; CNR, contrast to noise ratio; FFR, fractional flow reserve*.

No single technique appears to have a clear diagnostic advantage over other, and selection is primarily based on patient-specific criteria, local expertise, and technique availability. A number of studies examined the diagnostic performance of non-invasive stress tests for the detection of graft failure or indeed the progression of native coronary artery disease post CABG (Table 3). These reported variable diagnostic accuracy against invasive coronary angiography and the majority made no distinction between ischaemia secondary to graft failure or ischaemia secondary to non-grafted native vessel disease. Furthermore, the lack of baseline studies immediately or soon after surgery makes it difficult to draw conclusions about ischaemia caused by incomplete revascularisation at the time of surgery vs. ischaemia caused by a new pathophysiological process. Finally, most studies used a combination of symptomatic and asymptomatic patients, making comparisons between modalities challenging.

**Table 3:** Diagnostic performance of non-invasive stress tests to identify graft failure and native disease progression post coronary artery bypass graft surgery.

**References**	**Modality**	**Type of stress**	**Time from CABG (years)**	**Number of patients**	**Study population symptom status**	**Sensitivity (%)**	**Specificity (%)**
Pittella et al. (97)	Echocardiography	Dobutamine	0.32	25	Asymptomatic patients	83	69
Hoffman et al. (98)	Echocardiography	Dobutamine	6.4	60	Symptomatic [45] and asymptomatic [15] patients	78	86
Sawada et al. (99)	Echocardiography	Exercise	6.3	41	Symptomatic [23] and asymptomatic [18] patients	88	86
Chirillo et al. (100)	Echocardiography	Dipyridamole	2.2	106	Patients scheduled to undergo coronary angiography	67	91
Elhendy et al. (101)	Echocardiography	Dobutamine	5.1	60	Both symptomatic [38] and asymptomatic [12] patients	78	89
Kafka et al. (102)	Echocardiography	Exercise	3.6	182	Mostly asymptomatic patients [148]	77	96
Crouse et al. (116)	Echocardiography	Exercise	7	125	Mainly symptomatic patients [96]	98	92
Al Aloul et al. (88)	SPECT	Exercise	1	79	Unselected cohort prospectively assessed 1 year post CABG	77	69
Pfisterer et al. (107)	SPECT	Exercise	12	55	Symptomatic [26] and asymptomatic [29] patients	80	88
Khoury et al. (108)	SPECT	Adenosine	6.7	109	Wide range of indications for cohort selection, including “periodic check-up” in 31 patients	96	61
Lakkis et al. (109)	SPECT	Exercise	4.2	50	30 patients with typical and 20 patients with atypical chest pain	80	87
Klein et al. (96)	Perfusion CMR	Adenosine	8	78	Suspicion of progression of stable angina	77	90
Bernhardt et al. (57)	Perfusion CMR	Adenosine	1.2	110	Clinical indication for invasive angiography	73	77
Klein et al. (86)^*^	Perfusion CMR Dobutamine	Dobutamine (wall motion analysis)	9.5	109	Data not available	88	96

*SPECT, Single-Photon Emission Computed Tomography; CMR, Cardiac Magnetic Resonance; PET, Positron Emission Tomography*.

* *Abstract only*.

Comparison of the diagnostic accuracy of each test is hindered by the lack of systematic evaluation of their limitations in this patient group, but also the absence of contemporary studies using the latest state of the art tools. Indeed, most studies were historically performed using SPECT and stress echocardiography and were significantly limited by the existing technology, which warrants caution in extrapolating these results to current practice. Certainly, the true potential of modern tools of advanced echocardiography such as strain and myocardial contrast echocardiography (117), the use of solid-state detector technology in SPECT imaging (118) as well as artificial intelligence-based approaches in quantitative myocardial perfusion in the evaluation of patients with prior CABG remains unknown.

## Prognostic Role of Ischaemia Testing Following Surgical Revascularisation

Despite the technical challenges associated with ischaemia testing in patients with prior CABG surgery, a number of studies across the entire spectrum of imaging modalities suggested that detection of ischaemia post CABG predicts adverse clinical outcomes (Table 3). As such, evaluation of ischaemia in this group of patients becomes important for both risk stratification and for potentially guiding treatment decisions.

Historical data using exercise testing suggested that the presence of residual ischaemia post CABG is associated with increased risk of mortality, even among asymptomatic patients (119). Given the limitations of treadmill exercise ECG testing in patients with prior CABG (120), a number of studies subsequently evaluated the prognostic effect of ischaemia testing using non-invasive imaging, with the majority demonstrating a prognostic role for these tests (Table 4).

**Table 4:** Prognostic role of non-invasive ischaemia testing in patients with prior coronary artery bypass graft surgery.

References^*^	**Study design**	**Imaging modality**	**Stressor**	**Number of patients**	**Male (%)**	**Follow up (months)**	**Study result**
Cortigiani et al. (121)	Observational, multicenter	Stress echo	Dipyridamole	349	77	22	Ischemia associated with prognosis. CFVR of LAD ≤ 2 associated with HR 2.28
Harb et al. (122)	Observational, single center	Stress echo	Exercise	962	88	69	Ischaemia predicted mortality (HR 2.10)
Cortigiani et al. (123)	Observational, single center	Stress echo	Dobutamine Exercise Dipyridamole	500	80	25	Peak wall motion score index predicted mortality and MI (HR 3.07)
Arruda et al. (124)	Observational, single center	Stress echo	Exercise	718	82	35	18% reduction in hazard for every 10% incremental increase in exercise LVEF
Ortiz et al. (22)	Observational, single center	SPECT	Exercise Adenosine	84	100	119	Defect size 1 year following CABG, predicted death and CHF
Acampa et al. (110)	Observational, single center	SPECT	Dipyridamole Exercise	362	90	26	SPECT performed 5 years after CABG predicted death and MI (HR 3.7).
Sarda et al. (111)	Observational, single center	SPECT	Dipyridamole Exercise	115	90	35	Extent of stress defect predicted cardiac death and MI
Shapira et al. (112)^*^	Observational, single center	SPECT	-	170	-	48	SPECT performed soon after CABG has prognostic value
Palmas et al. (125)	Observational, single center	SPECT	Exercise	294	86	31	Incremental prognostic information provided by SPECT
Miller et al. (126)	Observational, single center	SPECT	Exercise	411	80	70	Exercise Tl-201 imaging performed within 2 years of CABG predicts outcomes
Lauer et al. (127)	Observational, single center	SPECT	Exercise	873	91	36	Exercise capacity and perfusion defects predict death (HR 2.78) in asymptomatic patients
Zellweger et al. (128)	Observational, single center	SPECT	Adenosine Exercise	1,765	80	23	MPS is strongly predictive of subsequent adverse events
Pen et al. (129)	Observational, multi-center	PET	Site-specific	953	70.8	29	Summed stress score predicted mortality (HR1.6) and cardiac death (HR1.8)
Kinnel et al. (130)	Observational, single center	CMR	Dipyridamole	852	89	50.4	Ischaemia predicted CV death (HR 2.15)

*SPECT, Single-Photon Emission Computed Tomography; CMR, Cardiac Magnetic Resonance; PET, Positron Emission Tomography; HR, hazard ratio; MI, myocardial infarction; CHF, Congestive heart failure*.

* *Abstract available only*.

Most evidence on the prognostic impact of ischaemia detection comes from stress echocardiography (121, 123, 124, 131) and SPECT imaging (22, 31, 110–112, 127, 128), reflecting the dominant role of these modalities, particularly in previous decades. Despite including a large number of patients and long follow up times, collective interpretation of these studies is made difficult by significant study design heterogeneity (131), including differences in stress agents, use of optimal medical therapy, abnormal test definitions and primary end points. Very few studies have used advanced imaging techniques (121), including newly developed methods of quantitative myocardial perfusion evaluation which have already demonstrated incremental prognostic utility in patients without prior CABG (118, 132–134). Furthermore, in view of their retrospective design most studies did not provide data on the mechanism of ischaemia, complicating the translation of this finding into a form of clinical therapy.

There is a wide range of pathophysiological processes that can contribute to myocardial ischaemia in patients post CABG, and each may have a different impact on prognosis. Graft failure, native disease progression and microvascular dysfunction may all affect patient outcomes, but their respective contribution is unclear. Similarly, outcomes following any form of revascularisation are generally thought to be superior if “complete revascularisation” is achieved, with a reduction in adverse events including subsequent myocardial infarction, repeat revascularisation and mortality (135). The mechanisms by which the completeness of revascularisation affects outcomes are also not well-defined, and may not be entirely associated with restoration of myocardial blood flow. One of the challenges in unraveling this, is that the vast majorities of studies have used relative crude anatomical definitions of completeness of revascularisation (136) with very few studies deploying a functional assessment for evaluating the effect of completeness of revascularisation on prognosis (137, 138).

However, evidence supporting the notion that detection of ischaemia in this patient group improves clinical outcomes is lacking, particularly in asymptomatic patients. Harb et al. (122), evaluated the impact of routine stress testing post CABG, and found that although ischaemia detection was associated with adverse clinical events, repeat revascularisation did not alter outcomes. Similarly, in the large, multi-centered ROSETTA-CABG registry, patients undergoing routine post-CABG perfusion assessment with SPECT were compared with patients undergoing selective testing, and no difference in adverse clinical outcomes between the two groups was found (139).

## Future Directions

The field of cardiac imaging has undergone dramatic developments in recent years, not only enabling enhanced diagnostic accuracy but providing tools for re-evaluating physiology and pathophysiological processes. Indeed, basic concepts in clinical cardiology, including that of myocardial ischaemia, continue to be centered on knowledge derived several decades ago and commonly remain uncontested. Recent studies, such as the ORBITA (140) and ISCHEMIA (141) trials have challenged our traditional ideas of myocardial ischaemia and its impact on patients symptoms and outcomes.

Advances in scanner performance, image reconstruction and wider availability of machine learning methods for data analysis have made it feasible to introduce quantitative methods of myocardial perfusion into routine clinical workflow. Such quantitative measures can be acquired in a highly automated fashion and offer incremental diagnostic value for ischaemia assessment particularly in complex models of coronary artery disease (142). Despite this, validation of such non-invasive myocardial perfusion indices against invasive coronary physiology in patients with prior CABG is lacking. Future prospective studies with paired information on coronary anatomy and quantitative perfusion imaging could provide new insights into the pathophysiological mechanisms of ischaemia in this patient population, potentially offering improved patient risk stratification and identification of novel therapeutic targets.

## Conclusion

**Figure 6:**
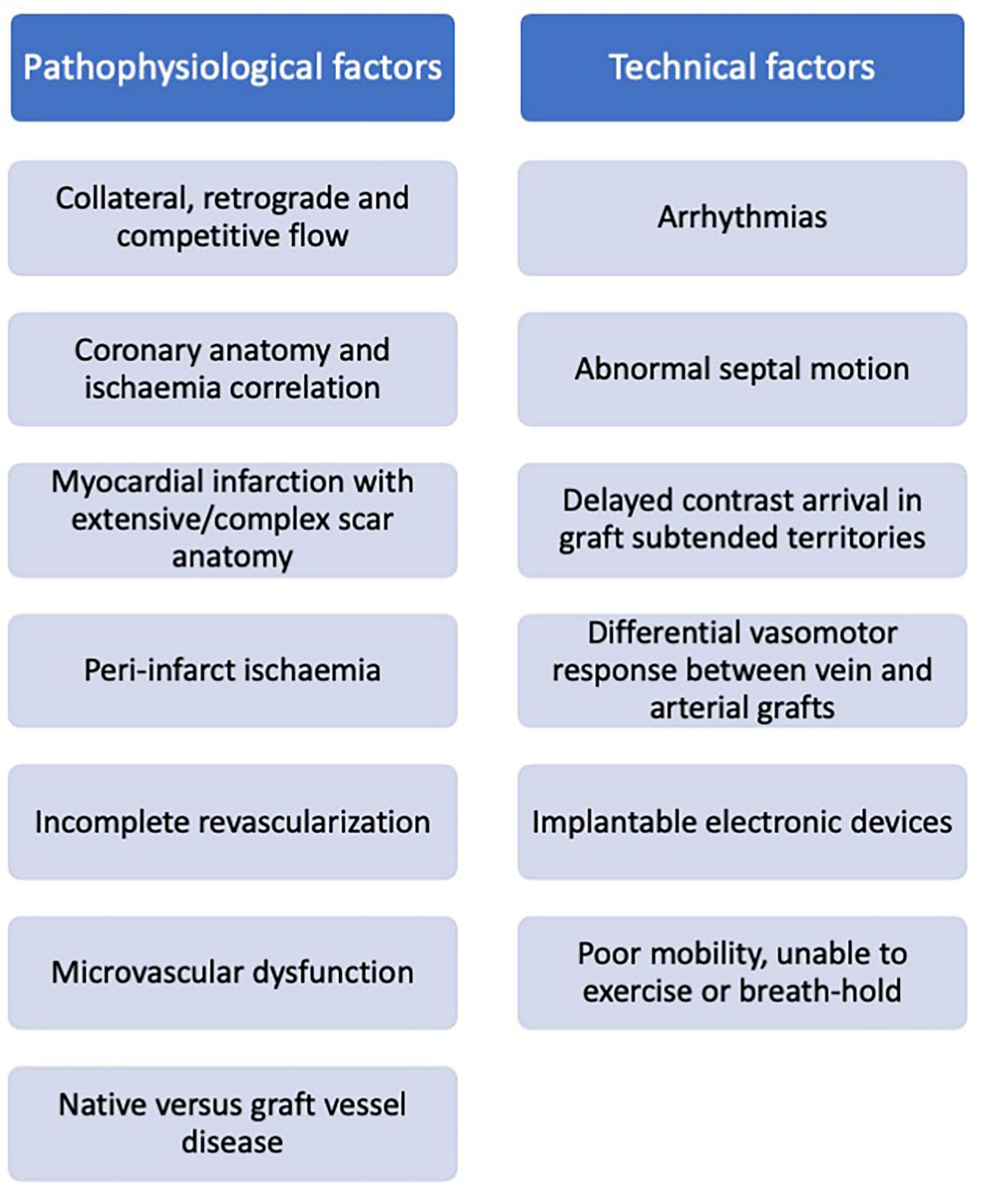
Factors impacting non-invasive ischaemia assessment in patients with prior surgical revascularization.

Patients commonly re-present for clinical assessment post coronary artery bypass grafting, and often pose a diagnostic challenge. Ischaemia evaluation in these patients is complex and subsequent clinical decision-making in response to the imaging results may be inconsistent. Challenges relate to both cardiovascular disease complexity (native coronary disease and collateralisation, graft variation, and infarction), and technical difficulties (arrhythmia, contrast transit time, and devices) (Figure 6), with no single imaging technique demonstrating clear superiority. Acknowledging the technical limitations of each modality may facilitate our decision making in selecting the most appropriate test based on the clinical scenario, personalized to the individual patient. Advances in imaging technology combined with the enhanced computational support of machine learning may help our understanding of these mechanisms, offering insights into the effects of revascularisation and potentially identifying novel therapeutic targets.

## Author Contributions

All authors listed have made a substantial, direct, and intellectual contribution to the work and approved it for publication.

## Funding

This work was supported by a Clinical Research Training Fellowship (A Seraphim) from the British Heart Foundation (FS/18/83/34025) and directly and indirectly from the NIHR Biomedical Research Centers at University College London Hospitals and Barts Health NHS Trusts. This work forms part of the research areas contributing to the translational research portfolio of the Biomedical Research Center at Barts which was supported and funded by the National Institute for Health Research.

## Conflict of Interest

The authors declare that the research was conducted in the absence of any commercial or financial relationships that could be construed as a potential conflict of interest.

## Publisher's Note

All claims expressed in this article are solely those of the authors and do not necessarily represent those of their affiliated organizations, or those of the publisher, the editors and the reviewers. Any product that may be evaluated in this article, or claim that may be made by its manufacturer, is not guaranteed or endorsed by the publisher.

## Abbreviations

ACC: American College of Cardiology
AHA: American Heart Association
CABG: Coronary Artery Bypass Graft
CMR: Cardiovascular Magnetic Resonance
CNR: Contrast to Noise Ratio
CTCA: Computed Tomography Coronary Angiography
FFR: Fractional Flow Reserve
LAD: Left Anterior Descending (artery)
LIMA: Left Internal Mammary Artery
LGE: Late Gadolinium Enhancement
LV: Left Ventricle
MACE: Major adverse cardiovascular events
MBF: Myocardial Blood Flow
MPR: Myocardial Perfusion Reserve
PCI: Percutaneous Coronary Intervention
PET: Positron Emission Tomography
SPECT: Single-Photon Emission Computed.

## References

[1] Weiss AJ Elixhauser A. Trends in operating room procedures in U.S. Hospitals, 2001–2011: statistical brief #171. In: Healthcare Cost and Utilization Project (HCUP) Statistical Briefs. Rockville: MD: Agency for Healthcare Research and Quality (US) (2006). Available online at: http://www.ncbi.nlm.nih.gov/books/NBK201926/ (accessed May 17, 2021).

[2] Yusuf S Zucker D Peduzzi P Fisher LD Takaro T Kennedy JW. Effect of coronary artery bypass graft surgery on survival: overview of 10-year results from randomised trials by the Coronary Artery Bypass Graft Surgery Trialists Collaboration. Lancet Lond Engl. (1994) 344:563–70. 10.1016/S0140-6736(94)91963-1 7914958

[3] The BARI 2D Study Group. A randomized trial of therapies for type 2 diabetes and coronary artery disease. N Engl J Med. (2009) 360:2503–15. 10.1056/NEJMoa0805796 19502645 PMC2863990

[4] Velazquez EJ Lee KL Jones RH Al-Khalidi HR Hill JA Panza JA. Coronary-artery bypass surgery in patients with ischemic cardiomyopathy. N Engl J Med. (2016) 374:1511–20. 10.1056/NEJMoa1602001 27040723 PMC4938005

[5] Fitzgibbon GM Kafka HP Leach AJ Keon WJ Hooper GD Burton JR. Coronary bypass graft fate and patient outcome: angiographic follow-up of 5,065 grafts related to survival and reoperation in 1,388 patients during 25 years. J Am Coll Cardiol. (1996) 28:616–26. 10.1016/0735-1097(96)00206-9 8772748

[6] Engebretsen KVT Friis C Sandvik L Tønnessen T. Survival after CABG–better than predicted by EuroSCORE and equal to the general population. Scand Cardiovasc J SCJ. (2009) 43:123–8. 10.1080/14017430802354085 18781451

[7] Adelborg K Horváth-Puhó E Schmidt M Munch T Pedersen L Nielsen PH. Thirty-year mortality after coronary artery bypass graft surgery: a Danish Nationwide Population-Based Cohort Study. Circ Cardiovasc Qual Outcomes. (2017) 10:e002708. 10.1161/CIRCOUTCOMES.116.002708 28500223

[8] Sergeant P Blackstone E Meyns B Stockman B Jashari R. First cardiological or cardiosurgical reintervention for ischemic heart disease after primary coronary artery bypass grafting. Eur J Cardio-Thorac Surg Off J Eur Assoc Cardio-Thorac Surg. (1998) 14:480–7. 10.1016/S1010-7940(98)00214-0 9860204

[9] Stone GW Kappetein AP Sabik JF Pocock SJ Morice M-C Puskas J. Five-year outcomes after PCI or CABG for left main coronary disease. N Engl J Med. (2019) 381:1820–30. 10.1056/NEJMoa1909406 31562798

[10] Lenzen MJ Boersma E Bertrand ME Maier W Moris C Piscione F. Management and outcome of patients with established coronary artery disease: the Euro Heart Survey on coronary revascularization. Eur Heart J. (2005) 26:1169–79. 10.1093/eurheartj/ehi238 15802360

[11] Rathod KS Beirne A-M Bogle R Firoozi S Lim P Hill J. Prior coronary artery bypass graft surgery and outcome after percutaneous coronary intervention: an observational study from the Pan-London Percutaneous Coronary Intervention Registry. J Am Heart Assoc. (2020) 9:e014409. 10.1161/JAHA.119.014409 32475202 PMC7429029

[12] Nikolsky E McLaurin BT Cox DA Manoukian SV Xu K Mehran R. Outcomes of patients with prior coronary artery bypass grafting and acute coronary syndromes: analysis from the ACUITY (Acute Catheterization and Urgent Intervention Triage Strategy) trial. JACC Cardiovasc Interv. (2012) 5:919–26. 10.1016/j.jcin.2012.06.009 22995879

[13] Björk VO Ekeström S Henze A Ivert T Landou C. Early and late patency of aortocoronary vein grafts. Scand J Thorac Cardiovasc Surg. (1981) 15:11–21. 10.3109/14017438109101020 6973814

[14] Lytle BW Loop FD Cosgrove DM Ratliff NB Easley K Taylor PC. Long-term (5 to 12 years) serial studies of internal mammary artery and saphenous vein coronary bypass grafts. J Thorac Cardiovasc Surg. (1985) 89:248–58. 10.1016/S0022-5223(19)38820-8 2857209

[15] Hess CN Lopes RD Gibson CM Hager R Wojdyla DM Englum BR. Saphenous vein graft failure after coronary artery bypass surgery: insights from PREVENT IV. Circulation. (2014) 130:1445–51. 10.1161/CIRCULATIONAHA.113.008193 25261549 PMC4206593

[16] Zhao Q Zhu Y Xu Z Cheng Z Mei J Chen X. Effect of ticagrelor plus aspirin, ticagrelor alone, or aspirin alone on saphenous vein graft patency 1 year after coronary artery bypass grafting: a randomized clinical trial. J Am Med Assoc. (2018) 319:1677–86. 10.1001/jama.2018.3197 29710164 PMC5933396

[17] Goldman S Zadina K Moritz T Ovitt T Sethi G Copeland JG. Long-term patency of saphenous vein and left internal mammary artery grafts after coronary artery bypass surgery: results from a Department of Veterans Affairs Cooperative Study. J Am Coll Cardiol. (2004) 44:2149–56. 10.1016/j.jacc.2004.08.064 15582312

[18] Sabik JF Lytle BW Blackstone EH Houghtaling PL Cosgrove DM. Comparison of saphenous vein and internal thoracic artery graft patency by coronary system. Ann Thorac Surg. (2005) 79:544–51. 10.1016/j.athoracsur.2004.07.047 15680832

[19] Pond KK Martin GV Every N Lehmann KG Anderson R Caldwell JH. Predictors of progression of native coronary narrowing to total occlusion after coronary artery bypass grafting. Am J Cardiol. (2003) 91:971–4. 10.1016/S0002-9149(03)00115-2 12686339

[20] Lopes RD Mehta RH Hafley GE Williams JB Mack MJ Peterson ED. Relationship between vein graft failure and subsequent clinical outcomes after coronary artery bypass surgery. Circulation. (2012) 125:749–56. 10.1161/CIRCULATIONAHA.111.040311 22238227 PMC3699199

[21] Loop FD Lytle BW Cosgrove DM Stewart RW Goormastic M Williams GW. Influence of the internal-mammary-artery graft on 10-year survival and other cardiac events. N Engl J Med. (1986) 314:1–6. 10.1056/NEJM198601023140101 3484393

[22] Ortiz F Mbai M Adabag S Garcia S Nguyen J Goldman S. Utility of nuclear stress imaging in predicting long-term outcomes one-year post CABG surgery. J Nucl Cardiol Off Publ Am Soc Nucl Cardiol. (2018) 2018:1469. 10.1007/s12350-018-01469-y 30397864

[23] Pellicano M De Bruyne B Toth GG Casselman F Wijns W Barbato E. Fractional flow reserve to guide and to assess coronary artery bypass grafting. Eur Heart J. (2017) 38:1959–68. 10.1093/eurheartj/ehw505 28025191

[24] Zimmermann FM Ferrara A Johnson NP van Nunen LX Escaned J Albertsson P. Deferral vs. performance of percutaneous coronary intervention of functionally non-significant coronary stenosis: 15-year follow-up of the DEFER trial. Eur Heart J. (2015) 36:3182–8. 10.1093/eurheartj/ehv452 26400825

[25] Escaned J. Secondary revascularization after CABG surgery. Nat Rev Cardiol. (2012) 9:540–9. 10.1038/nrcardio.2012.100 22776987

[26] Trägårdh E Tan SS Bucerius J Gimelli A Gaemperli O Lindner O. Systematic review of cost-effectiveness of myocardial perfusion scintigraphy in patients with ischaemic heart disease: a report from the cardiovascular committee of the European Association of Nuclear Medicine. Endorsed by the European Association of Cardiovascular Imaging. Eur Heart J Cardiovasc Imaging. (2017) 18:825–32. 10.1093/ehjci/jex095 28549119

[27] National Institute of Clinical Excellence. Recent-Onset Chest Pain of Suspected Cardiac Origin: Assessment and Diagnosis. (2016). p. 30. Available online at: https://www.nice.org.uk/guidance/cg95 (accessed December 4, 2021). 32065740

[28] Knuuti J Wijns W Saraste A Capodanno D Barbato E Funck-Brentano C. 2019 ESC Guidelines for the diagnosis and management of chronic coronary syndromes. Eur Heart J. (2020) 41:407–77. 10.1093/eurheartj/ehz425 31504439

[29] merican College of Cardiology Foundation Appropriate Use Criteria Task Force, American Society of Echocardiography, American Heart Association, American Society of Nuclear Cardiology, Heart Failure Society of America, Heart Rhythm Society. ACCF/ASE/AHA/ASNC/HFSA/HRS/SCAI/SCCM/SCCT/SCMR 2011 appropriate use criteria for echocardiography. a report of the American College of Cardiology Foundation Appropriate Use Criteria Task Force, American Society of Echocardiography, American Heart Association, American Society of Nuclear Cardiology, Heart Failure Society of America, Heart Rhythm Society, Society for Cardiovascular Angiography and Interventions, Society of Critical Care Medicine, Society of Cardiovascular Computed Tomography, Society for Cardiovascular Magnetic Resonance American College of Chest Physicians. J Am Soc Echocardiogr Off Publ Am Soc Echocardiogr. (2011) 24:229–67. 10.1016/j.echo.2010.12.008 21338862

[30] Fihn SD Gardin JM Abrams J Berra K Blankenship JC Dallas AP. 2012 ACCF/AHA/ACP/AATS/PCNA/SCAI/STS guideline for the diagnosis and management of patients with stable ischemic heart disease. J Am Coll Cardiol. (2012) 60:e44–164. 10.1016/j.jacc.2012.07.012 23182125

[31] Eisenberg MJ Wou K Nguyen H Duerr R Del Core M Fourchy D. Use of stress testing early after coronary artery bypass graft surgery. Am J Cardiol. (2006) 97:810–6. 10.1016/j.amjcard.2005.09.130 16516581

[32] Ellenberger C Sologashvili T Cikirikcioglu M Verdon G Diaper J Cassina T. Risk factors of postcardiotomy ventricular dysfunction in moderate-to-high risk patients undergoing open-heart surgery. Ann Card Anaesth. (2017) 20:287–96. 10.4103/aca.ACA_60_17 28701592 PMC5535568

[33] Ding W Ji Q Shi Y Ma R. Predictors of low cardiac output syndrome after isolated coronary artery bypass grafting. Int Heart J. (2015) 56:144–9. 10.1536/ihj.14-231 25740396

[34] Wilson RF Marcus ML White CW. Effects of coronary bypass surgery and angioplasty on coronary blood flow and flow reserve. Prog Cardiovasc Dis. (1988) 31:95–114. 10.1016/0033-0620(88)90013-8 2971242

[35] Coolong A Baim DS Kuntz RE O'Malley AJ Marulkar S Cutlip DE. Saphenous vein graft stenting and major adverse cardiac events: a predictive model derived from a pooled analysis of 3958 patients. Circulation. (2008) 117:790–7. 10.1161/CIRCULATIONAHA.106.651232 18212287

[36] Pucelikova T Mehran R Kirtane AJ Kim Y-H Fahy M Weisz G. Short- and long-term outcomes after stent-assisted percutaneous treatment of saphenous vein grafts in the drug-eluting stent era. Am J Cardiol. (2008) 101:63–8. 10.1016/j.amjcard.2007.07.048 18157967

[37] Morrison DA Sethi G Sacks J Henderson WG Grover F Sedlis S. Percutaneous coronary intervention versus repeat bypass surgery for patients with medically refractory myocardial ischemia: AWESOME randomized trial and registry experience with post-CABG patients. J Am Coll Cardiol. (2002) 40:1951–4. 10.1016/S0735-1097(02)02560-3 12475454

[38] Harskamp RE Beijk MA Damman P Kuijt WJ Woudstra P Grundeken MJ. Clinical outcome after surgical or percutaneous revascularization in coronary bypass graft failure. J Cardiovasc Med Hagerstown Md. (2013) 14:438–45. 10.2459/JCM.0b013e328356a4fc 22828774

[39] Berry C Pieper KS White HD Solomon SD Van de Werf F Velazquez EJ. Patients with prior coronary artery bypass grafting have a poor outcome after myocardial infarction: an analysis of the VALsartan in acute myocardial iNfarcTion trial (VALIANT). Eur Heart J. (2009) 30:1450–6. 10.1093/eurheartj/ehp102 19346225

[40] Brown LAE Saunderson CED Das A Craven T Levelt E Knott KD. A comparison of standard and high dose adenosine protocols in routine vasodilator stress cardiovascular magnetic resonance: dosage affects hyperaemic myocardial blood flow in patients with severe left ventricular systolic impairment. J Cardiovasc Magn Reson. (2021) 23:37. 10.1186/s12968-021-00714-7 33731141 PMC7971951

[41] Geleijnse ML Krenning BJ Nemes A van Dalen BM Soliman O ten Cate FJ. Incidence, pathophysiology, and treatment of complications during dobutamine-atropine stress echocardiography. Circulation. (2010) 121:1756–67. 10.1161/CIRCULATIONAHA.109.859264 20404267

[42] Lim CC Tan CS Chia CML Tan AK Choo JCJ Kaushik M. Long-term risk of progressive chronic kidney disease in patients with severe acute kidney injury requiring dialysis after coronary artery bypass surgery. Cardiorenal Med. (2015) 5:157–63. 10.1159/000381068 26195967 PMC4478312

[43] Maaniitty T Jaakkola S Saraste A Knuuti J. Hybrid coronary computed tomography angiography and positron emission tomography myocardial perfusion imaging in evaluation of recurrent symptoms after coronary artery bypass grafting. Eur Heart J - Cardiovasc Imaging. (2019) 20:1298–304. 10.1093/ehjci/jey160 30388202

[44] Kawai H Sarai M Motoyama S Ito H Takada K Harigaya H. A combination of anatomical and functional evaluations improves the prediction of cardiac event in patients with coronary artery bypass. BMJ Open. (2013) 3:e003474. 10.1136/bmjopen-2013-003474 24220113 PMC3831107

[45] Nathoe Hendrik M Erik B Jansen Erik WL Suyker Willem JL Stella Pieter R Lahpor Jaap R. Role of coronary collaterals in off-pump and on-pump coronary bypass surgery. Circulation. (2004) 110:1738–42. 10.1161/01.CIR.0000143105.42988.FD 15381650

[46] Guo L Steinman DA Moon BC Wan W-K Millsap RJ. Effect of distal graft anastomosis site on retrograde perfusion and flow patterns of native coronary vasculature. Ann Thorac Surg. (2001) 72:782–7. 10.1016/S0003-4975(01)02801-6 11565658

[47] Zafrir N Madduri J Mats I Ben-Gal T Solodky A Assali A. Discrepancy between myocardial ischemia and luminal stenosis in patients with left internal mammary artery grafting to left anterior descending coronary artery. J Nucl Cardiol Off Publ Am Soc Nucl Cardiol. (2003) 10:663–8. 10.1016/j.nuclcard.2003.09.003 14668779

[48] Henzlova MJ Bittner V Dubovsky E Nath H Pohost GM. Frequency of stress-induced thallium-201 defects in patients with patent internal mammary artery to the left anterior descending coronary artery graft. Am J Cardiol. (1992) 70:399–400. 10.1016/0002-9149(92)90631-8 1632415

[49] Hirzel HO Nuesch K Sialer G Horst W Krayenbuehl HP. Thallium-201 exercise myocardial imaging to evaluate myocardial perfusion after coronary artery bypass surgery. Br Heart J. (1980) 43:426–35. 10.1136/hrt.43.4.426 6967325 PMC482304

[50] Wainwright RJ Brennand-Roper DA Maisey MN Sowton E. Exercise thallium-201 myocardial scintigraphy in the follow-up of aortocoronary bypass graft surgery. Br Heart J. (1980) 43:56–66. 10.1136/hrt.43.1.56 6965585 PMC482242

[51] Seraphim A Knott KD Beirne A-M Augusto JB Menacho K Artico J. Use of quantitative cardiovascular magnetic resonance myocardial perfusion mapping for characterization of ischemia in patients with left internal mammary coronary artery bypass grafts. J Cardiovasc Magn Reson. (2021) 23:82. 10.1186/s12968-021-00763-y 34134696 PMC8210347

[52] Spyrou N Khan MA Rosen SD Foale R Davies DW Sogliani F. Persistent but reversible coronary microvascular dysfunction after bypass grafting. Am J Physiol Heart Circ Physiol. (2000) 279:H2634–40. 10.1152/ajpheart.2000.279.6.H2634 11087215

[53] Kelle S Graf K Dreysse S Schnackenburg B Fleck E Klein C. Evaluation of contrast wash-in and peak enhancement in adenosine first pass perfusion CMR in patients post bypass surgery. J Cardiovasc Magn Reson Off J Soc Cardiovasc Magn Reson. (2010) 12:28. 10.1186/1532-429X-12-28 20465836 PMC2887852

[54] Janiec M Nazari Shafti TZ Dimberg A Lagerqvist B Lindblom RPF. Graft failure and recurrence of symptoms after coronary artery bypass grafting. Scand Cardiovasc J SCJ. (2018) 52:113–9. 10.1080/14017431.2018.1442930 29508655

[55] Pegg TJ Selvanayagam JB Francis JM Karamitsos TD Maunsell Z Yu L-M. A randomized trial of on-pump beating heart and conventional cardioplegic arrest in coronary artery bypass surgery patients with impaired left ventricular function using cardiac magnetic resonance imaging and biochemical markers. Circulation. (2008) 118:2130–8. 10.1161/CIRCULATIONAHA.108.785105 18981306

[56] Steuer J Bjerner T Duvernoy O Jidéus L Johansson L Ahlström H. Visualisation and quantification of peri-operative myocardial infarction after coronary artery bypass surgery with contrast-enhanced magnetic resonance imaging. Eur Heart J. (2004) 25:1293–9. 10.1016/j.ehj.2004.05.015 15288156

[57] Bernhardt P Spiess J Levenson B Pilz G Höfling B Hombach V. Combined assessment of myocardial perfusion and late gadolinium enhancement in patients after percutaneous coronary intervention or bypass grafts: a multicenter study of an integrated cardiovascular magnetic resonance protocol. JACC Cardiovasc Imaging. (2009) 2:1292–300. 10.1016/j.jcmg.2009.05.011 19909933

[58] Schnell F Donal E Bernard A Thebault C Lelong B Kervio G. Improved diagnosis of post-operative myocardial infarction by contrast echocardiography after coronary artery bypass graft surgery. Eur J Echocardiogr. (2011) 12:612–8. 10.1093/ejechocard/jer087 21785121

[59] Almeida AG Carpenter J-P Cameli M Donal E Dweck MR Flachskampf FA. Multimodality imaging of myocardial viability: an expert consensus document from the European Association of Cardiovascular Imaging (EACVI). Eur Heart J - Cardiovasc Imaging. (2021) 22:e97–125. 10.1093/ehjci/jeab053 34097006

[60] Neumann F-J Sousa-Uva M Ahlsson A Alfonso F Banning AP Benedetto U. 2018 ESC/EACTS Guidelines on myocardial revascularization. Eur Heart J. (2019) 40:87–165. 10.1093/eurheartj/ehy855 30165437

[61] Brown JM Poston RS Gammie JS Cardarelli MG Schwartz K Sikora JAH. Off-pump versus on-pump coronary artery bypass grafting in consecutive patients: decision-making algorithm and outcomes. Ann Thorac Surg. (2006) 81:555–61. 10.1016/j.athoracsur.2005.06.081 16427851

[62] Garcia S Sandoval Y Roukoz H Adabag S Canoniero M Yannopoulos D. Outcomes after complete versus incomplete revascularization of patients with multivessel coronary artery disease: a meta-analysis of 89,883 patients enrolled in randomized clinical trials and observational studies. J Am Coll Cardiol. (2013) 62:1421–31. 10.1016/j.jacc.2013.05.033 23747787

[63] Aikawa T Naya M Koyanagawa K Manabe O Obara M Magota K. Improved regional myocardial blood flow and flow reserve after coronary revascularization as assessed by serial 15O-water positron emission tomography/computed tomography. Eur Heart J Cardiovasc Imaging. (2020) 21:36–46. 10.1093/ehjci/jez220 31544927

[64] Driessen RS Danad I Stuijfzand WJ Schumacher SP Knuuti J Mäki M. Impact of revascularization on absolute myocardial blood flow as assessed by serial [15O]H_2_O positron emission tomography imaging: a comparison with fractional flow reserve. Circ Cardiovasc Imaging. (2018) 11:e007417. 10.1161/CIRCIMAGING.117.007417 29703779

[65] Gould KL Johnson NP Bateman TM Beanlands RS Bengel FM Bober R. Anatomic versus physiologic assessment of coronary artery disease. Role of coronary flow reserve, fractional flow reserve, and positron emission tomography imaging in revascularization decision-making. J Am Coll Cardiol. (2013) 62:1639–53. 10.1016/j.jacc.2013.07.076 23954338

[66] Brown LAE Onciul SC Broadbent DA Johnson K Fent GJ Foley JRJ. Fully automated, inline quantification of myocardial blood flow with cardiovascular magnetic resonance: repeatability of measurements in healthy subjects. J Cardiovasc Magn Reson. (2018) 20:48. 10.1186/s12968-018-0462-y 29983119 PMC6036695

[67] Zorach B Shaw PW Bourque J Kuruvilla S Balfour PC Yang Y. Quantitative cardiovascular magnetic resonance perfusion imaging identifies reduced flow reserve in microvascular coronary artery disease. J Cardiovasc Magn Reson. (2018) 20:14. 10.1186/s12968-018-0435-1 29471856 PMC5822618

[68] Rahman H Scannell CM Demir OM Ryan M McConkey H Ellis H. High-resolution cardiac magnetic resonance imaging techniques for the identification of coronary microvascular dysfunction. JACC Cardiovasc Imaging. (2020) 10:15. 10.1016/j.jcmg.2020.10.015 33248969

[69] Nishi T Murai T Ciccarelli G Shah SV Kobayashi Y Derimay F. Prognostic value of coronary microvascular function measured immediately after percutaneous coronary intervention in stable coronary artery disease. Circ Cardiovasc Interv. (2019) 12:e007889. 10.1161/CIRCINTERVENTIONS.119.007889 31525096

[70] Sellke FW Friedman M Dai HB Shafique T Schoen FJ Weintraub RM. Mechanisms causing coronary microvascular dysfunction following crystalloid cardioplegia and reperfusion. Cardiovasc Res. (1993) 27:1925–32. 10.1093/cvr/27.11.1925 8287398

[71] Akasaka T Yoshikawa J Yoshida K Maeda K Hozumi T Nasu M. Flow capacity of internal mammary artery grafts: early restriction and later improvement assessed by Doppler guide wire. Comparison with saphenous vein grafts. J Am Coll Cardiol. (1995) 25:640–7. 10.1016/0735-1097(94)00448-Y 7860908

[72] Feng J Liu Y Chu LM Singh AK Dobrilovic N Fingleton JG. Changes in microvascular reactivity after cardiopulmonary bypass in patients with poorly controlled versus controlled diabetes. Circulation. (2012) 126(11Suppl.1):S73–80. 10.1161/CIRCULATIONAHA.111.084590 22965996 PMC3448935

[73] Kotecha T Martinez-Naharro A Boldrini M Knight D Hawkins P Kalra S. Automated pixel-wise quantitative myocardial perfusion mapping by CMR to detect obstructive coronary artery disease and coronary microvascular dysfunction: validation against invasive coronary physiology. JACC Cardiovasc Imaging. (2019) 12:1958–69. 10.1016/j.jcmg.2018.12.022 30772231 PMC8414332

[74] Kellman P Hansen MS Nielles-Vallespin S Nickander J Themudo R Ugander M. Myocardial perfusion cardiovascular magnetic resonance: optimized dual sequence and reconstruction for quantification. J Cardiovasc Magn Reson. (2017) 19:43. 10.1186/s12968-017-0355-5 28385161 PMC5383963

[75] Arnold JR Francis JM Karamitsos TD Lim CC van Gaal WJ Testa L. Myocardial perfusion imaging after coronary artery bypass surgery using cardiovascular magnetic resonance: a validation study. Circ Cardiovasc Imaging. (2011) 4:312–8. 10.1161/CIRCIMAGING.110.959742 21343329

[76] Sommer K Bernat D Schmidt R Breit H-C Schreiber LM. Resting myocardial blood flow quantification using contrast-enhanced magnetic resonance imaging in the presence of stenosis: a computational fluid dynamics study. Med Phys. (2015) 42:4375–84. 10.1118/1.4922708 26133634

[77] Martens J Panzer S van den Wijngaard JPHM Siebes M Schreiber LM. Analysis of coronary contrast agent transport in bolus-based quantitative myocardial perfusion MRI measurements with computational fluid dynamics simulations. In: Pop M Wright GA, editors, Functional Imaging and Modelling of the Heart. Cham: Springer International Publishing (2017). p. 369–80. 10.1007/978-3-319-59448-4_35

[78] Layland J Carrick D Lee M Oldroyd K Berry C. Adenosine: physiology, pharmacology, and clinical applications. JACC Cardiovasc Interv. (2014) 7:581–91. 10.1016/j.jcin.2014.02.009 24835328

[79] Chong WC Collins P Webb CM De Souza AC Pepper JR Hayward CS. Comparison of flow characteristics and vascular reactivity of radial artery and long saphenous vein grafts [NCT00139399]. J Cardiothorac Surg. (2006) 1:4. 10.1186/1749-8090-1-4 16722590 PMC1440301

[80] Glineur D Poncelet A El Khoury G D'hoore W Astarci P Zech F. Fractional flow reserve of pedicled internal thoracic artery and saphenous vein grafts 6 months after bypass surgery. Eur J Cardio-Thorac Surg Off J Eur Assoc Cardio-Thorac Surg. (2007) 31:376–81. 10.1016/j.ejcts.2006.11.023 17174100

[81] Hanet C Robert A Wijns W. Vasomotor response to ergometrine and nitrates of saphenous vein grafts, internal mammary artery grafts, and grafted coronary arteries late after bypass surgery. Circulation. (1992) 86(5Suppl.):II210–6. 10.1016/0735-1097(91)91805-O 1424002

[82] Ochiai M Ohno M Taguchi J Hara K Suma H Isshiki T. Responses of human gastroepiploic arteries to vasoactive substances: comparison with responses of internal mammary arteries and saphenous veins. J Thorac Cardiovasc Surg. (1992) 104:453–8. 10.1016/S0022-5223(19)34803-2 1495310

[83] Arnold JR Karamitsos TD van Gaal WJ Testa L Francis JM Bhamra-Ariza P. Residual ischemia after revascularization in multivessel coronary artery disease: insights from measurement of absolute myocardial blood flow using magnetic resonance imaging compared with angiographic assessment. Circ Cardiovasc Interv. (2013) 6:237–45. 10.1161/CIRCINTERVENTIONS.112.000064 23696598

[84] Fung AY Gallagher KP Buda AJ. The physiologic basis of dobutamine as compared with dipyridamole stress interventions in the assessment of critical coronary stenosis. Circulation. (1987) 76:943–51. 10.1161/01.CIR.76.4.943 3652428

[85] Geleijnse ML Elhendy A Fioretti PM Roelandt JRTC. Dobutamine stress myocardial perfusion imaging. J Am Coll Cardiol. (2000) 36:2017–27. 10.1016/S0735-1097(00)01012-3 11127435

[86] Klein C Gebker R Kelle S Graf K Dreysse S Schnackenburg B. Direct comparison of CMR dobutamine stress wall motion and perfusion analysis with adenosine perfusion in patients after bypass surgery. J Cardiovasc Magn Reson. (2011) 13:P100. 10.1186/1532-429X-13-S1-P100

[87] Manka R Jahnke C Gebker R Schnackenburg B Paetsch I. Head-to-head comparison of first-pass MR perfusion imaging during adenosine and high-dose dobutamine/atropine stress. Int J Cardiovasc Imaging. (2011) 27:995–1002. 10.1007/s10554-010-9748-3 21088993

[88] Al Aloul B Mbai M Adabag S Garcia S Thai H Goldman S. Utility of nuclear stress imaging for detecting coronary artery bypass graft disease. BMC Cardiovasc Disord. (2012) 12:62. 10.1186/1471-2261-12-62 22862805 PMC3469356

[89] Hatam N Aljalloud A Mischke K Karfis EA Autschbach R Hoffmann R. Interatrial conduction disturbance in postoperative atrial fibrillation: a comparative study of P-wave dispersion and Doppler myocardial imaging in cardiac surgery. J Cardiothorac Surg. (2014) 9:114. 10.1186/1749-8090-9-114 24957051 PMC4082174

[90] Reynolds HR Tunick PA Grossi EA Dilmanian H Colvin SB Kronzon I. Paradoxical septal motion after cardiac surgery: a review of 3,292 cases. Clin Cardiol. (2007) 30:621–3. 10.1002/clc.20201 18069678 PMC6653372

[91] Eslami B Roitman D Karp RB Sheffield LT. The echocardiogram after pericardiectomy. Jpn Heart J. (1979) 20:1–5. 10.1536/ihj.20.1 449039

[92] Thielmann M Sharma V Al-Attar N Bulluck H Bisleri G Bunge JJ. ESC Joint Working Groups on Cardiovascular Surgery and the Cellular Biology of the Heart Position Paper: peri-operative myocardial injury and infarction in patients undergoing coronary artery bypass graft surgery. Eur Heart J. (2017) 38:2392–411. 10.1093/eurheartj/ehx383 28821170 PMC5808635

[93] D'Agostino RS Jacobs JP Badhwar V Fernandez FG Paone G Wormuth DW. The society of thoracic surgeons adult cardiac surgery database: 2018 update on outcomes and quality. Ann Thorac Surg. (2018) 105:15–23. 10.1016/j.athoracsur.2017.10.035 29233331

[94] Lehmann KG Lee FA McKenzie WB Barash PG Prokop EK Durkin MA. Onset of altered interventricular septal motion during cardiac surgery. Assessment by continuous intraoperative transesophageal echocardiography. Circulation. (1990) 82:1325–34. 10.1161/01.CIR.82.4.1325 2401066

[95] Duncan A Francis D Gibson D Pepper J Henein M. Electromechanical left ventricular resynchronisation by coronary artery bypass surgery. Eur J Cardio-Thorac Surg Off J Eur Assoc Cardio-Thorac Surg. (2004) 26:711–9. 10.1016/j.ejcts.2004.05.020 15450561

[96] Klein C Nagel E Gebker R Kelle S Schnackenburg B Graf K. Magnetic resonance adenosine perfusion imaging in patients after coronary artery bypass graft surgery. JACC Cardiovasc Imaging. (2009) 2:437–45. 10.1016/j.jcmg.2008.12.016 19580726

[97] Pittella FJM Cunha AB da Romeu Filho LJM Labrunie MM Weitzel LH Melhado JC. Functional assessment of coronary grafts on dobutamine pharmacological stress echocardiogram. Arq Bras Cardiol. (2006) 87:451–5. 10.1590/S0066-782X2006001700009 17128314

[98] Hoffmann R Lethen H Falter F Flachskampf FA Hanrath P. Dobutamine stress echocardiography after coronary artery bypass grafting. Transthoracic vs. biplane transoesophageal imaging. Eur Heart J. (1996) 17:222–9. 10.1093/oxfordjournals.eurheartj.a014838 8732375

[99] Sawada SG Judson WE Ryan T Armstrong WF Feigenbaum H. Upright bicycle exercise echocardiography after coronary artery bypass grafting. Am J Cardiol. (1989) 64:1123–9. 10.1016/0002-9149(89)90864-3 2683711

[100] Chirillo F Bruni A De Leo A Olivari Z Franceschini-Grisolia E Totis O. Usefulness of dipyridamole stress echocardiography for predicting graft patency after coronary artery bypass grafting. Am J Cardiol. (2004) 93:24–30. 10.1016/j.amjcard.2003.09.007 14697461

[101] Elhendy A Geleijnse ML Roelandt JR Cornel JH van Domburg RT El-Refaee M. Assessment of patients after coronary artery bypass grafting by dobutamine stress echocardiography. Am J Cardiol. (1996) 77:1234–6. 10.1016/S0002-9149(96)00171-3 8651104

[102] Kafka H Leach AJ FitzGibbon GM. Exercise echocardiography after coronary artery bypass surgery: correlation with coronary angiography. J Am Coll Cardiol. (1995) 25:1019–23. 10.1016/0735-1097(94)00532-U 7897111

[103] Senior R Lepper W Pasquet A Chung G Hoffman R Vanoverschelde J-L. Myocardial perfusion assessment in patients with medium probability of coronary artery disease and no prior myocardial infarction: comparison of myocardial contrast echocardiography with 99mTc single-photon emission computed tomography. Am Heart J. (2004) 147:1100–5. 10.1016/j.ahj.2003.12.030 15199362

[104] Nagel E Greenwood JP McCann GP Bettencourt N Shah AM Hussain ST. Magnetic resonance perfusion or fractional flow reserve in coronary disease. N Engl J Med. (2019) 380:2418–28. 10.1056/NEJMoa1716734 31216398

[105] Kwong RY Ge Y Steel K Bingham S Abdullah S Fujikura K. Cardiac magnetic resonance stress perfusion imaging for evaluation of patients with chest pain. J Am Coll Cardiol. (2019) 74:1741–55. 10.1016/j.jacc.2019.07.074 31582133 PMC8109181

[106] Bhuva AN Moralee R Brunker T Lascelles K Cash L Patel KP. Evidence to support magnetic resonance conditional labelling of all pacemaker and defibrillator leads in patients with cardiac implantable electronic devices. Eur Heart J. (2021) 2021:ehab350. 10.1093/eurheartj/ehab350 34435642 PMC9259370

[107] Pfisterer M Emmenegger H Schmitt HE Müller-Brand J Hasse J Grädel E. Accuracy of serial myocardial perfusion scintigraphy with thallium-201 for prediction of graft patency early and late after coronary artery bypass surgery. A controlled prospective study. Circulation. (1982) 66:1017–24. 10.1161/01.CIR.66.5.1017 6982112

[108] Khoury AF Rivera JM Mahmarian JJ Verani MS. Adenosine thallium-201 tomography in evaluation of graft patency late after coronary artery bypass graft surgery. J Am Coll Cardiol. (1997) 29:1290–5. 10.1016/S0735-1097(97)00045-4 9137226

[109] Lakkis NM Mahmarian JJ Verani MS. Exercise thallium-201 single photon emission computed tomography for evaluation of coronary artery bypass graft patency. Am J Cardiol. (1995) 76:107–11. 10.1016/S0002-9149(99)80039-3 7611141

[110] Acampa W Petretta M Evangelista L Nappi G Luongo L Petretta MP. Stress cardiac single-photon emission computed tomographic imaging late after coronary artery bypass surgery for risk stratification and estimation of time to cardiac events. J Thorac Cardiovasc Surg. (2008) 136:46–51. 10.1016/j.jtcvs.2007.10.011 18603052

[111] Sarda L Fuchs L Lebtahi R Faraggi M Delahaye N Hvass U. Prognostic value of 201Tl myocardial scintigraphy after coronary artery bypass grafting. Nucl Med Commun. (2001) 22:189–96. 10.1097/00006231-200102000-00011 11258406

[112] Shapira I Heller I Kornizky Y Topilsky M Isakov A. The value of stress thallium-201 single photon emission CT imaging as a predictor of outcome and long-term prognosis after CABG. J Med. (2001) 32:271–82. 11958274

[113] Weustink AC Nieman K Pugliese F Mollet NR Meijboom WB Meijboom BW. Diagnostic accuracy of computed tomography angiography in patients after bypass grafting: comparison with invasive coronary angiography. JACC Cardiovasc Imaging. (2009) 2:816–24. 10.1016/j.jcmg.2009.02.010 19608130

[114] Eisenberg C Hulten E Bittencourt MS Blankstein R. Use of CT angiography among patients with prior coronary artery bypass grafting surgery. Cardiovasc Diagn Ther. (2017) 7:102–5. 10.21037/cdt.2016.11.08 28164019 PMC5253442

[115] Nørgaard BL Fairbairn TA Safian RD Rabbat MG Ko B Jensen JM. Coronary CT angiography-derived fractional flow reserve testing in patients with stable coronary artery disease: recommendations on interpretation and reporting. Radiol Cardiothorac Imaging. (2019) 1:e190050. 10.1148/ryct.2019190050 33778528 PMC7977999

[116] Crouse LJ Vacek JL Beauchamp GD Porter CB Rosamond TL Kramer PH. Exercise echocardiography after coronary artery bypass grafting. Am J Cardiol. (1992) 70:572–6. 10.1016/0002-9149(92)90193-3 1510004

[117] Toulemonde MEG Corbett R Papadopoulou V Chahal N Li Y Leow CH. High frame-rate contrast echocardiography: in-human demonstration. JACC Cardiovasc Imaging. (2018) 11:923–4. 10.1016/j.jcmg.2017.09.011 29248652

[118] Otaki Y Betancur J Sharir T Hu L-H Gransar H Liang JX. 5-year prognostic value of quantitative versus visual MPI in subtle perfusion defects: results from REFINE SPECT. JACC Cardiovasc Imaging. (2020) 13:774–85. 10.1016/j.jcmg.2019.02.028 31202740 PMC6899217

[119] Weiner DA Ryan TJ Parsons L Fisher LD Chaitman BR Sheffield LT. Prevalence and prognostic significance of silent and symptomatic ischemia after coronary bypass surgery: a report from the Coronary Artery Surgery Study (CASS) randomized population. J Am Coll Cardiol. (1991) 18:343–8. 10.1016/0735-1097(91)90584-V 1856402

[120] Krone RJ Hardison RM Chaitman BR Gibbons RJ Sopko G Bach R. Risk stratification after successful coronary revascularization: the lack of a role for routine exercise testing. J Am Coll Cardiol. (2001) 38:136–42. 10.1016/S0735-1097(01)01312-2 11451263

[121] Cortigiani L Ciampi Q Rigo F Bovenzi F Picano E Sicari R. Prognostic value of dual imaging stress echocardiography following coronary bypass surgery. Int J Cardiol. (2019) 277:266–71. 10.1016/j.ijcard.2018.09.105 30292434

[122] Harb SC Cook T Jaber WA Marwick TH. Exercise testing in asymptomatic patients after revascularization: are outcomes altered? Arch Intern Med. (2012) 172:854–61. 10.1001/archinternmed.2012.1355 22905351

[123] Cortigiani L Bigi R Sicari R Landi P Bovenzi F Picano E. Stress echocardiography for the risk stratification of patients following coronary bypass surgery. Int J Cardiol. (2010) 143:337–42. 10.1016/j.ijcard.2009.03.063 19342111

[124] Arruda AM McCully RB Oh JK Mahoney DW Seward JB Pellikka PA. Prognostic value of exercise echocardiography in patients after coronary artery bypass surgery. Am J Cardiol. (2001) 87:1069–73. 10.1016/S0002-9149(01)01463-1 11348604

[125] Palmas W Bingham S Diamond GA Denton TA Kiat H Friedman JD. Incremental prognostic value of exercise thallium-201 myocardial single-photon emission computed tomography late after coronary artery bypass surgery. J Am Coll Cardiol. (1995) 25:403–9. 10.1016/0735-1097(94)00380-9 7829794

[126] Miller TD Christian TF Hodge DO Mullan BP Gibbons RJ. Prognostic value of exercise thallium-201 imaging performed within 2 years of coronary artery bypass graft surgery. J Am Coll Cardiol. (1998) 31:848–54. 10.1016/S0735-1097(98)00011-4 9525558

[127] Lauer MS Lytle B Pashkow F Snader CE Marwick TH. Prediction of death and myocardial infarction by screening with exercise-thallium testing after coronary-artery-bypass grafting. Lancet Lond Engl. (1998) 351:615–22. 10.1016/S0140-6736(97)07062-1 9500316

[128] Zellweger MJ Lewin HC Lai S Dubois EA Friedman JD Germano G. When to stress patients after coronary artery bypass surgery? Risk stratification in patients early and late post-CABG using stress myocardial perfusion SPECT: implications of appropriate clinical strategies. J Am Coll Cardiol. (2001) 37:144–52. 10.1016/S0735-1097(00)01104-9 11153729

[129] Pen A Yam Y Chen L Dorbala S Di Carli MF Merhige ME. Prognostic value of Rb-82 positron emission tomography myocardial perfusion imaging in coronary artery bypass patients. Eur Heart J Cardiovasc Imaging. (2014) 15:787–92. 10.1093/ehjci/jet259 24477784

[130] Kinnel M Sanguineti F Pezel T Unterseeh T Hovasse T Toupin S. Prognostic value of vasodilator stress perfusion CMR in patients with previous coronary artery bypass graft. Eur Heart J Cardiovasc Imaging. (2020) 2020:jeaa316. 10.1016/j.acvdsp.2020.10.056 33313780

[131] Harb SC Marwick TH. Prognostic value of stress imaging after revascularization: a systematic review of stress echocardiography and stress nuclear imaging. Am Heart J. (2014) 167:77–85. 10.1016/j.ahj.2013.07.035 24332145

[132] Knott KD Seraphim A Augusto JB Xue H Chacko L Aung N. The prognostic significance of quantitative myocardial perfusion: an artificial intelligence based approach using perfusion mapping. Circulation. (2020) 119:44666. 10.1161/CIRCULATIONAHA.119.044666 32078380 PMC7176346

[133] Juárez-Orozco LE Tio RA Alexanderson E Dweck M Vliegenthart R El Moumni M. Quantitative myocardial perfusion evaluation with positron emission tomography and the risk of cardiovascular events in patients with coronary artery disease: a systematic review of prognostic studies. Eur Heart J Cardiovasc Imaging. (2018) 19:1179–87. 10.1093/ehjci/jex331 29293983 PMC6148746

[134] Patel KK Spertus JA Chan PS Sperry BW Al Badarin F Kennedy KF. Myocardial blood flow reserve assessed by positron emission tomography myocardial perfusion imaging identifies patients with a survival benefit from early revascularization. Eur Heart J. (2020) 41:759–68. 10.1093/eurheartj/ehz389 31228200 PMC7828468

[135] Gaba P Gersh BJ Ali ZA Moses JW Stone GW. Complete versus incomplete coronary revascularization: definitions, assessment and outcomes. Nat Rev Cardiol. (2021) 18:155–68. 10.1038/s41569-020-00457-5 33067581

[136] Vander Salm TJ Kip KE Jones RH Schaff HV Shemin RJ Aldea GS. What constitutes optimal surgical revascularization? Answers from the bypass angioplasty revascularization investigation (BARI). J Am Coll Cardiol. (2002) 39:565–72. 10.1016/S0735-1097(01)01806-X 11849852

[137] Thuesen AL Riber LP Veien KT Christiansen EH Jensen SE Modrau I. Fractional flow reserve versus angiographically-guided coronary artery bypass grafting. J Am Coll Cardiol. (2018) 72:2732–43. 10.1016/j.jacc.2018.09.043 30497559

[138] Fournier S Toth GG De Bruyne B Johnson NP Ciccarelli G Xaplanteris P. Six-year follow-up of fractional flow reserve-guided versus angiography-guided coronary artery bypass graft surgery. Circ Cardiovasc Interv. (2018) 11:e006368. 10.1161/CIRCINTERVENTIONS.117.006368 29848611

[139] ROSETTA-CABG Registry Eisenberg MJ Wou K Nguyen H Duerr R Del Core M. Lack of benefit for routine functional testing early after coronary artery bypass graft surgery: results from the ROSETTA-CABG Registry. J Invasive Cardiol. (2006) 18:147–52. 16729399

[140] Al-Lamee R Thompson D Dehbi H-M Sen S Tang K Davies J. Percutaneous coronary intervention in stable angina (ORBITA): a double-blind, randomised controlled trial. Lancet Lond Engl. (2018) 391:31–40. 10.1016/j.jvs.2017.11.046 29103656

[141] Maron DJ Hochman JS Reynolds HR Bangalore S O'Brien SM Boden WE. Initial invasive or conservative strategy for stable coronary disease. N Engl J Med. (2020) 382:1395–407. 10.1056/NEJMoa1915922 32227755 PMC7263833

[142] Kotecha T Chacko L Chehab O O'Reilly N Martinez-Naharro A Lazari J. Assessment of multivessel coronary artery disease using cardiovascular magnetic resonance pixelwise quantitative perfusion mapping. JACC Cardiovasc Imaging. (2020) 13:2546–57. 10.1016/j.jcmg.2020.06.041 33011115

